# Digital Cognitive Biomarker for Mild Cognitive Impairments and Dementia: A Systematic Review

**DOI:** 10.3390/jcm11144191

**Published:** 2022-07-19

**Authors:** Zihan Ding, Tsz-lok Lee, Agnes S. Chan

**Affiliations:** 1 Neuropsychology Laboratory, Department of Psychology, The Chinese University of Hong Kong, Hong Kong, China; cameliading@link.cuhk.edu.hk (Z.D.); tllee@link.cuhk.edu.hk (T.-l.L.); 2 Research Centre for Neuropsychological Well-Being, The Chinese University of Hong Kong, Hong Kong, China

**Keywords:** digital biomarker, digital cognitive biomarker, computerized test, digital cognitive test, dementia, mild cognitive impairment, systematic review

## Abstract

The dementia population is increasing as the world’s population is growing older. The current systematic review aims to identify digital cognitive biomarkers from computerized tests for detecting dementia and its risk state of mild cognitive impairment (MCI), and to evaluate the diagnostic performance of digital cognitive biomarkers. A literature search was performed in three databases, and supplemented by a Google search for names of previously identified computerized tests. Computerized tests were categorized into five types, including memory tests, test batteries, other single/multiple cognitive tests, handwriting/drawing tests, and daily living tasks and serious games. Results showed that 78 studies were eligible. Around 90% of the included studies were rated as high quality based on the Newcastle–Ottawa Scale (NOS). Most of the digital cognitive biomarkers achieved comparable or even better diagnostic performance than traditional paper-and-pencil tests. Moderate to large group differences were consistently observed in cognitive outcomes related to memory and executive functions, as well as some novel outcomes measured by handwriting/drawing tests, daily living tasks, and serious games. These outcomes have the potential to be sensitive digital cognitive biomarkers for MCI and dementia. Therefore, digital cognitive biomarkers can be a sensitive and promising clinical tool for detecting MCI and dementia.

## 1. Introduction

The incidence and prevalence of dementia increase as the world’s population is growing older. It may partially be due to the growing incidence of age-related comorbidities such as hypertension and glycemic variability, as they are risk factors for cognitive impairments and dementia [1,2]. According to World Health Organization (WHO) global status report on the public health response to dementia [3], the dementia population will grow from 55 million in 2019 to 78 million in 2030 and will raise to 139 million in 2050. The fast increase in the dementia population results in a growing burden on families and healthcare systems. It was estimated that the annual worldwide cost of dementia is over USD 1.3 trillion, and will reach USD 2.8 trillion by 2030 [3]. Nevertheless, many people live with undiagnosed or delayed diagnoses. One study analyzed the rate of undiagnosed dementia based on a subsample (*n* = 598) of a large epidemiological cohort study on older adults aged ≥65 years in England [4]. In 2011–2013, only 43% of the people who met the dementia criteria received diagnoses in primary care, and the average time from meeting the criteria to diagnosis was 3.5 years. Several barriers to dementia diagnosis were reported, such as the complexity of traditional clinical diagnosis [5], limited access to diagnostic services especially for people living in less developed areas [6], and a lack of awareness of non-memory cognitive impairments in people living with dementia and their informants as well as in clinical diagnosis [4].

Mild cognitive impairment (MCI) was identified as a transitional stage between normal aging and dementia [7], and the amnestic type of MCI (aMCI) was especially risky of progressing into dementia [8]. On average, about 30% of people with MCI progressed to dementia [9]. Compared with providing interventions at the diagnosis of moderate Alzheimer’s disease (AD), early detection and interventions of MCI prolongs years of high functioning with the disease [10]. One review showed that cognitive training serves as a preventive method for older adults at risk of cognitive decline [11]. Therefore, early detection of dementia and MCI can be critical for early interventions and planning for the future, so as to delay cognitive deterioration and improve quality of life [12].

Digital biomarkers may help to tackle the existing problems in detecting MCI and dementia, and contribute to the early detection. The widespread availability of digital devices (e.g., computers, tablets, smartphones, wearables) enables easy access to the measurement of cognition and behaviors, or even continuous monitoring. Digital biomarkers are defined as objective, quantifiable physiological and behavioral data obtained via digital devices that can be used to predict and interpret health outcomes [13]. According to another definition provided by U.S. Food and Drug Administration (FDA), digital biomarker is “a characteristic or set of characteristics” that can be assessed through digital health technologies, and serves as an indicator for normal biological processes, pathogenic processes, and therapeutic or intervention response [14].

Two types of tasks for yielding digital biomarkers were identified: one is active tasks that require active engagement in the evaluation, such as computerized versions of traditional cognitive tests (e.g., [15,16]) or online games (e.g., [17,18,19]) for assessing a broad domain of cognitive functions, voluntary speech production for assessing language ability [20], or virtual reality techniques (e.g., [21,22]). Another type is passive tasks in which people were monitored without active input, such sleep quality, walking speed, phone usage, and daily activities [13,23]. Among the different types of methods and platforms for assessing digital biomarkers, computerized tests seem to be especially accessible for detecting MCI and dementia by measuring the cognitive functions that are potentially impaired.

A recent systematic review on digital cognitive tests reported that most of the digital cognitive tests showed comparable diagnostic performance with paper-and-pencil tests in MCI and dementia [24]. Since many of the digital cognitive tests are test batteries that use the composite of performance in multiple subsets to detect MCI and dementia, we are interested in whether some subsets may be more sensitive than other subsets in detecting MCI or dementia from normal healthy adults. Therefore, the present systematic review aims to identify potential sensitive digital cognitive biomarkers yielded from computerized tests for detecting MCI and dementia. There are two main research questions: (1) Which cognitive outcomes of computerized tests showed significantly large group differences between MCI and normal adults or between dementia and normal adults, indicating they have the potential to become sensitive digital cognitive biomarkers for MCI and dementia? (2) How is the diagnostic performance of the digital cognitive biomarkers?

## 2. Methods

### 2.1. Criteria for Study Inclusion and Exclusion

The study selection was based on the following inclusion criteria: (1) the topic is on computerized tests or digital biomarkers based on computerized tests for differentiating and/or diagnosing MCI and dementia from normal healthy adults; (2) containing a group of normal health adults as the control group, and a clinical group of MCI and/or dementia; (3) the diagnosis of clinical groups should be confirmed in accordance with standardized diagnostic criteria, including but not limited to: the Clinical Dementia Rating (CDR) [25], any versions of the Diagnostic and Statistical Manual of Mental Disorder (e.g., [26]), the recommendations from National Institute on Ageing-Alzheimer’s Association on the diagnosis for Alzheimer’s disease [27], the Petersen criterion [28], standardized cognitive or neuropsychological tests, or diagnosis from a certificated physician, neurologist, or geriatric psychiatrist based on various measurements such as brain imaging and blood test; (4) the study adopted one or more computerized tests as a measurement tool, including computerized tests developed or adapted from traditional cognitive or neuropsychological tests, computerized version of novel experimental paradigms, and online games, that can be accessed simply through computers, tablets, and smart phones; (5) the study should be empirical research published in journals and written in English.

Moreover, the exclusion criteria include: (1) no control group and/or clinical groups of MCI or dementia; (2) diagnosis criteria for clinical groups were not reported; (3) only used measuring techniques that cannot be simply accessed through computers, tablets, and smart phones, such as neuroimaging techniques, eye-tracking, immersive virtual reality and passive monitoring techniques; (4) used computer-simulated data or animal data; (5) article types other than empirical studies, such as literature reviews, conference abstracts, comments, case-reports, or study protocols.

### 2.2. Search Methods

The present systematic review followed the Preferred Reporting Items for Systematic Reviews and Meta-Analysis (PRISMA) statement (2020) [29]. A systematic literature search was performed on three databases including Scopus, PubMed, and Embase from March to April 2022, retrieving papers published from inception to 23 April 2022. The following search query was applied for searching in titles and abstracts of the papers: (“MCI” OR “mildly cognitive” OR “mild cognitive impairment” OR dement* OR Alzheimer*) AND (“digital biomarkers” OR digital OR telemonitoring OR e-health OR phone OR computer OR tablet OR wearable OR sensor) AND (assessment OR monitor* OR screen* OR detect* OR predict* OR diagnos* OR classif*). We also conducted a Google search for the names of computerized tests that were frequently mentioned in the previous reviews, including Cambridge Neuropsychological Test Automated Battery (CANTAB), BrainCheck, Smart Aging Serious Game (SASG), CogState, Mild Cognitive Impairment Screen (MCIS), CNS Vital Signs, Mindtreams, Cognitive Stability Index (CSI), and Computer-Administered Neuropsychological Screen for Mild Cognitive Impairment (CANS-MCI). Moreover, filters were used to only include journal articles published in English.

### 2.3. Selection of Studies

The search results were exported to EndNote software (version X9.3.3; Clarivate, Philadelphia, PA, USA), and were screened by the first author. Duplicates were removed both via the automatic duplicate finding function in the Endnote and through eyeball screening. The remaining unique items were initially screened on titles and abstracts and then reviewed on full text to decide the final eligibility.

### 2.4. Data Collection

The first author extracted data from each eligible study in the following domains: (1) demographical information, including sample size, gender, age, educational level, race or ethnicity, and diagnostic criteria for each group; (2) characteristics of the computerized test, including test name, test type or cognitive abilities measured by the test, original paper-and-pencil test (if any), administration time, devices used for data collection, and whether needs supervision from an experimenter during the test; (3) names of the cognitive outcomes measured by computerized tests; (4) quantitative results for calculating effect sizes for significant between-group mean difference in the cognitive outcomes measured by computerized tests, including mean, standard deviation (SD) or standard error (SE), and *p*-values; (5) diagnostic performance of the digital cognitive biomarkers from computerized tests and comparison paper-and-pencil tests (if any), including sensitivity, specificity, and area under the curve (AUC). In addition, PlotDigitizer (2022) [30] was used to extract data from figures when the data were presented only in figures rather than in tables or in main texts.

### 2.5. Data Analysis

Hedges’ *g* and 95% confidence intervals were calculated for estimating effect sizes of between-group mean difference [31]. When the sample size is smaller than 20, Hedges’ *g* provides a superior estimation of a mean difference than Cohen’s *d*. When the sample size is larger than 20, the mean difference estimation based on Hedges’ *g* and Cohen’s *d* is similar. Moreover, Helges’ *g* also outperforms Cohen’s d when group sizes are unequal. Considering that the sample sizes of our selected studies vary from less than 10 participants in a group to thousands of participants in a group, and the group size was highly unequal within some studies, Hedges’ *g* was preferred over Cohen’s *d* in estimating the standardized mean difference in the current review. For the interpretation of group differences, a value at 0.2 for Hedges’ *g* indicates a small effect, and a value at 0.5 and 0.8 correspond to moderate and large effects, respectively, according to the rule of thumb from Cohen [32]. A Hedges’ *g* larger than zero indicates that control group outperformed the other group. Hedges’ *g* was transformed to absolute value if a negative Hedges’ *g* indicates better performance in normal controls than MCI/dementia group.

Meta-analysis methods were not applicable for synthesizing data from different studies to provide a single effect estimation for group comparison (e.g., MCI versus control, dementia vs. control). This was because most of the selected studies have multiple measurement outcomes (i.e., multiple digital biomarkers for differentiating between MC/dementia and normal controls) within each study, and the correlations among these outcomes were not accessible from the data reported by most of the studies. Therefore, we could not simply combine the group mean difference in different outcomes for each study by calculating means or composite scores. Moreover, the experimental paradigms used in the computerized tests were heterogeneous, which does not approve of the interpretation of combining group mean differences across different studies as well. In this case, a systematic review summarizing the results would be preferred.

Group means difference (Hedges’ *g* and 95% confidence interval) for each group comparison were visualized using forest plots (Supplementary Figures S1–S3), and were summarized in Table 1. Diagnostic performance between two groups (e.g., MCI vs. control, dementia vs. control), including sensitivity, specificity, and AUC were presented in structured tables (Supplementary Tables S3–S5) and summarized in Table 2. The diagnostic performance of standardized, traditional paper-and-pencil tests was also included to examine whether the digital biomarkers yielded from computerized tests have comparable diagnostic accuracy to golden-standard tests.

### 2.6. Quality Assessment

Quality assessment of each study was conducted by the first author using The Newcastle–Ottawa Scale (NOS) for assessing the quality of case–control studies [33]. Each study was rated on nine questions from three domains, including the selection of cases and controls, comparability of cases and controls based on the design or analysis, and exposure. Considering that age and education level are potential confounders of cognitive functions, comparability was evaluated based on whether these two variables were matched between groups or controlled in statistical analysis. A total quality score was obtained for each study, with a maximum of 9. Studies that obtained a total quality score between 7 and 9 were regarded as having high quality, and studies that obtained a total quality score smaller than 7 were regarded as having high risk. Results were presented in Supplementary Table S6.

## 3. Results

### 3.1. Literature Search and Study Selection

**Figure 1:**
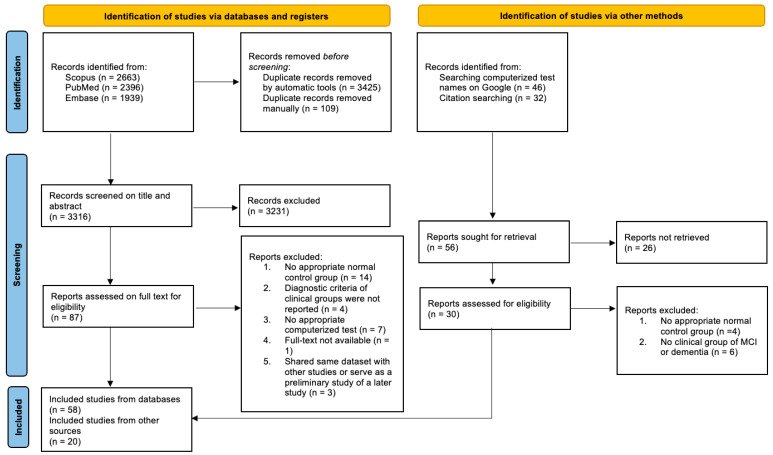
PRISMA flow diagram of study selection process.

After removing the duplicates, a total sample size of 3316 papers was obtained for screening titles and abstracts. The PRISMA flow diagram (Figure 1) shows the step-by-step paper selection. After an initial screening of the titles and abstracts, 87 studies were retrieved for full-text reviewing. It is worth noting that, among the 87 studies, 1 study by Ursenbach and colleagues [34] was excluded because it was analyzed on the same dataset as a study by Saxton and colleagues [35] and did not report any novel findings related to our research question. Moreover, an earlier study by Müller and colleagues [36] was a preliminary study of a later study [37]; therefore, only the results of the later study were included for analysis. Similarly, 1 study by Valladares-Rodriguez and colleagues [38] was also a preliminary study of a later study [39]; we only kept the later one. Another 2 studies by Gielis and colleagues [17,18] worked on the same dataset but provided findings related to different aspects of our research question. Therefore, according to the recommendation of Cochrane Reviews [40], both studies were included and combined for the results for further analysis.

After excluding the studies without a control group, diagnostic criteria for clinical groups, real data from humans, and appropriate computerized tests, there were 58 studies finally assessed from the database. An additional 6 papers were identified from references lists of previous studies, and 14 papers were identified through searching names of computerized tests in Google, resulting in a total sample of 78 studies for the systematic review.

### 3.2. Demographics of the Included Studies

Supplementary Table S1 shows the demographical characteristics of the included 78 studies. The group size ranged from 8 to 3263, and most of the studies have a group size smaller than 100. The mean age of participants in the control group ranged from 53.0 to 82.6 years (median = 72.5), the MCI group ranged from 55.0 to 85.2 years (median = 74.8), and the dementia group ranged from 58.0 to 82.1 years (median = 76.4). The mean education years of our participants range from 1.3 to 17.4 (median = 14.2), 1.8 to 16.7 (median = 13.1), and 1.8 to 21.3 (median = 12.2) for the control group, MCI group, and dementia group, respectively. In total, 16 studies reported the ethnicity or race of the sample, among which 13 studies recruited a sample mainly consisting of White or Caucasian (proportion range of White or Caucasian: 69–100%), 2 studies recruited a sample mixing different races such as Hispanic and non-Hispanic White and Black, Asian, and other races, and 1 study recruited a sample only consisting of Asian participants.

In addition to the MCI group and dementia group, another clinical group, termed as cognitively impaired (CI) group was also identified. This group was assumed to cover individuals with different levels of cognitive impairments ranging from mild cognitive impairments to different levels of dementia. The classification of CI group was either indicated by a standardized test such as CDR rating larger than 0.5, or indicated by the study results combining MCI and Dementia group as a separate CI group, and then compared or diagnosed the CI group against the control group.

### 3.3. Characteristics of the Computerized Tests

Computerized tests used in 78 selected studies can be classified into five categories (Supplementary Table S2). First of all, there were 10 studies using a computerized version of traditional memory tests (e.g., paired-associative learning (PAL), The Placing Test). Moreover, 37 studies used computerized test batteries consisting of multiple tasks translated from traditional tests or test batteries. Each test battery can cover a broad range of cognitive functions such as executive function, verbal and non-verbal memory, processing speed, language, and visuospatial skills. Thirdly, 12 studies adopted other single or multiple tests for various cognitive functions other than memory and cannot be regarded as a test battery, such as attention (Attentional Matrices Test (AMT)), psychomotor speed (Trail Making Test Part A and B (TMT-A and TMT-B)), reaction time (Simple Reaction Time Task), emotion task (Emotion Recognition Task), spatial neglect (Cancellation Test), navigation (Hidden Goal Task), as well as multiple cognitive functions in one single task (e.g., Rey-Osterrieth (Rey-O) Complex Figure). Another type of computerized test used in 10 studies can be described as digital handwriting or drawing tests, either based on traditional, well-established tests (e.g., digital Clock Drawing Test, digital Spiral Drawing Test), or novel tests (e.g., digital Chinese Handwriting task). Lastly, 9 studies reported novel tests, including computer simulations of daily living tasks (e.g., shopping, using an Automated Teller Machine (ATM), finding a way home, and medication management) and serious games (e.g., computer-based Klondike Solitaire card game, video games to assessing episodic memory).

The level of supervision was reported if related information was provided in the study. If the computerized tests required an experimenter to give instructions and assist participants to understand the task requirement, this computerized test was regarded as “supervised”. Minimal supervision was reported if a participant can self-administer the test, with minimal help from a family member or caregivers on the technical problems, or when the experimenter only stands by without any intrusion. The computerized test was regarded as unsupervised if participants can self-administer at home without any help. There were 7 studies that reported minimal supervision, 1 study reported minimal supervision or unsupervised administration, 1 study reported being supervised by a virtual assistant, and 6 studies reported unsupervised administration.

### 3.4. Group Difference in Cognitive Outcomes of Computerized Tests

We reported the group means difference of cognitive outcomes measured by computerized tests, and diagnostic performance of digital cognitive biomarkers in differentiating MCI and/or dementia from normal controls based on a 3 (three group comparisons: MCI vs. control, dementia vs. control, and CI vs. control) × 5 (five categories of computerized tests: memory test, test battery, other single/multiple cognitive tests, handwriting/drawing tests, and daily living tasks and serious games) structure.

#### 3.4.1. MCI vs. Control

As shown in Table 1, for the group comparison between MCI and Control, Helges’ *g* for memory tests ranged between 0.7 and 1.6, suggesting that memory-related outcomes differed largely between MCI and control. For computerized test batteries, the Hedges’ *g* varied from 0.1 to 2.9. Test batteries including CANTAB [41], computerized Beijing version of Montreal Cognitive Assessment (MoCA-CC) [42], CogState [43], and Brain Health Assessment (BHA) [44] were able to yield large group mean differences, especially in subsets measuring memory and executive functions. Cognitive outcomes of other single/multiple tests achieved a Hedges’ *g* range between 0.7 and 1.2. Correct cancellations in e-CT [45], performance in inspection time (IT) task [46], and digital Trail Making Test (dTMT) completion time [47] were found to yield the largest group difference, suggesting that processing speed, visual attention, and task switching ability were also able to differentiate MCI from normal controls.

Handwriting or drawing tests also showed variable group differences between MCI and normal controls (*g*: 0.1–2.1). Group differences were very large in three outcomes measured in digital Clock Drawing Test (dCDT), including time on surface (*g* = 2.1), time in air (*g* = 1.9), and total completion time (*g* = 1.1) [37]. Moreover, features of Chinese handwriting accuracy (i.e., stroke position control, stroke length, stroke orientation, and the ratio of in-air to on-paper trajectory length) also showed a very large difference between the two groups (*g* > 1.0) [48]. The other features in dCDT [37] and the digital Tree Drawing Test (dTDT) [49] only showed small to moderate differences between groups.

Most of the outcomes measured in serious games showed moderate to large differences between MCI and controls. Average thinking time (*g* = 1.3), average accuracy (*g* = 1.0), standard deviation of total time (*g* = 0.8) in Klondike Solitaire [17], and completion time of “Flipping cards” in Computerized Touch-Panel Games (*g* = 0.9) [50] showed large group differences. Moreover, for computer-simulated daily activity tasks, the total completion time of SIMulation-Based Assessment of Cognition (SIMBAC) also reported a large group difference (*g* = 0.9) [51].

#### 3.4.2. Dementia vs. Control

For computerized memory tests, the adjusted total errors in the PAL subset from CANTAB [52] and the performance in the Placing Test showed large group differences (*g* = 4.9 and *g* = 2.5, respectively) between dementia and controls [53]. Moreover, similar to the situation in comparing MCI with normal controls, the group mean difference in computerized test batteries varied largely from 0.3 to 7.2. While most of the cognitive outcomes measured in computerized test batteries showed moderate to large group differences, the visuospatial subset of Tablet-based Cognitive Assessments (*g* = 0.3) only showed small group difference [54]. Again, large group differences were reported in test batteries including CANTAB [41], NeuroTrax Mindstreams [55], Inbrain CST [15], and BHA [44], and the largest group differences were observed in memory subsets, followed by executive function subsets. Other single/multiple cognitive tests showed a large group means difference ranging from 0.9 to 3.1, suggesting that various cognitive domains including processing speed, attention, emotion recognition, and visual-motor skills can well-differentiate MCI from normal controls. Performance in computerized Simple Reaction Time (SRT) task and Flanker Response Time (FRT) (*g* = 3.1) [56], as well as correct cancellations in the electronic version of Cancellation Test (e-CT) (*g* = 2.3) [45] can best differentiate between MCI and normal controls.

Group means difference in outcomes measured in handwriting or drawing tests varied from 0.2 to 2.4. Similar to the results in comparing MCI with normal controls, several outcomes in the dCDT (i.e., total completion time, time in the air, minute hand distance from the center in command trial, hour hand distance from the center in copy trial) [57] and the Chinese Handwriting task (i.e., stroke position control, pause time per stroke, stroke orientation) [48] showed large group differences (*g* > 0.8).

Daily activity tasks and serious games showed a large group difference in differentiating Dementia from normal controls (*g*: 0.8–2.9). Both total accuracy (*g* = 2.9) and total completion time (*g* = 1.3) in SIMBAC can largely differentiated between dementia and normal controls [51]. Moreover, Computerized Touch-Panel Games that involve processing speed, recent and remote memory, attention, and executive functions also largely differentiated between dementia and normal controls [50].

#### 3.4.3. Cognitively Impaired (CI) vs. Control

When comparing CI with controls, digital outcomes from Trail-Making Test (TMT) including total score, total completion time, and total response time showed large group differences ranging between 1.0 and 2.0 [58]. Again, test batteries showed group differences that varied largely (*g*: 0.2–2.1). Test batteries including BHA [44], Computerized Dementia Screening Test (CDST) [59], Computerized Cognitive Screen (CoCoSc) [60], ComBased-CAT [61], and Boston Cognitive Assessment (BoCA) [62] showed large group differences, especially for subsets measuring memory, attention, and working memory. Moreover, performance in daily living tasks (i.e., Computer-based functional skills assessment and training (CFSAT)) showed moderate to large group differences (*g*: 0.7–1.2) [63].

### 3.5. Diagnostic Information

#### 3.5.1. MCI

The diagnostic performances were displayed in Supplementary Tables S3 and S4, and summarized in Table 2. For detecting MCI, digital biomarkers of memory tests achieved sensitivity between 42.0% and 85.8%, specificity between 66.0% and 93.3%, and AUC between 0.53 and 0.93. The best and second-best diagnostic performances for MCI were provided by two computerized memory tests based on the paired-associate learning (PAL) test [52,64]. Among these two memory tests, The Miami Test of Semantic Interference (MITSI) achieved a sensitivity of 85.5%, specificity of 84.4%, and an AUC of 0.93 [64], and the CANTAB-PAL showed an AUC of 0.80 [52]. Moreover, test batteries showed a variable diagnostic performance (sensitivity: 41.4–100.0%, specificity: 64.0–100.0%, AUC: 0.66–0.97).

Thirty-three (94%) digital cognitive biomarkers from the test batteries showed good diagnostic performance with sensitivity and specificity higher than 75%, or AUC larger than 0.7. Several test batteries provided excellent diagnostic performance with AUC close to or larger than 0.9, including BHA [44,65], Brain on Track Self-applied Computerized Test (BoT) [66], and MoCA-CC [42]. Other single/multiple cognitive tests obtained a sensitivity range of 56.3–84.7%, a specificity range of 53.6–90.5%, and an AUC range of 0.67–0.91. The best diagnosis was provided by the performance in digital version of Rey-O delay recall (sensitivity = 84.7%, specificity = 90.5%, AUC = 0.91) [67]. Handwriting/drawing tests showed sensitivity between 65.0% and 100.0%, specificity between 56.0% to 100.0%, and AUC between 0.77 and 0.89. Most of the digital biomarkers from handwriting/drawing tasks also yielded good diagnostic results. A discriminant function using kinematic and pressure features of the Spiral Drawing Test even yielded 100% sensitivity and 100% specificity [68]. However, it should be noted that the sample size of this study was relatively small, with 17 participants in the control group and 12 participants in the MCI group. Therefore, the diagnostic performance may be inflated. For the daily living tasks and serious games, the sensitivities were good (76.9–84.4%); specificity ranged between 58.0% and 88.9%, and AUC ranged between 0.77 and 0.90. The machine learning model using digital biomarkers of cognitive performance in Klondike Solitaire task provide a reasonable diagnostic performance (sensitivity = 77.78%, specificity = 88.89%, AUC = 0.90) [18].

#### 3.5.2. Dementia

For diagnosing dementia, memory tests (i.e., digital version of The Placing Test) showed good diagnostic performance (sensitivity = 88.9%, specificity = 92.9%) [53]. For test batteries, the diagnostic performance ranged varied largely (sensitivity: 52.9–100.0%, specificity: 56.0–100.0%, AUC: 0.54–0.99). Several test batteries provided excellent diagnostic performance with AUC larger than 0.9, including BHA [44,65], Computerized Self-Test (CST) [69], Computerized cognitive screening (CCS) [70], CANS-MCI [71], electronic version of Self-Administered Gerocognitive Examination (e-SAGE) [72], Computerized Screening Test Battery [73]. For other single/multiple cognitive tests, the sensitivity range was 62.7–86.1%, the specificity range was 75.0–95.3%, and the AUC range was 0.76–0.95, suggesting an overall satisfactory diagnostic performance. Similar to the diagnosis of MCI, the best diagnosis was provided by the performance in a digital version of Rey-O immediate recall (sensitivity = 82.7%, specificity = 94.7%, AUC = 0.95) [67]. Digital biomarkers of handwriting/drawing tests also yielded good diagnosis performance, with specificity ranging between 82.0% and 97.7%, and specificity ranging between 71.4% and 86.0%. For daily living tasks, SIMulation-Based Assessment of Cognition (SIMBAC) [51] achieved a good diagnostic performance, with a sensitivity of 86.0%, specificity of 75.0%, and an outstanding AUC of 0.97.

#### 3.5.3. Cognitively Impaired (CI)

Only one memory test reported diagnostic performance on CI, which was the Mild Cognitive Impairment Screen (MCIS) [74], a digital version of the Word List Memory subset within National Institute of Aging’s Consortium to Establish a Registry for Alzheimer’s Disease (CERAD) test battery [75]. MCIS showed good diagnostic performance, with sensitivity of 91.8%, specificity of 72.0%, and AUC of 0.89. Digital biomarkers based on test batteries also provided good diagnostic performance, with a sensitivity range of 70.7% to 91.0%, specificity range of 69.0% to 94.2%, and AUC range of 0.78 to 0.95. Overall performance in BHA [44] yielded the best diagnostic performance (sensitivity = 91.0%, specificity = 85.0%, AUC = 0.95) among all the test batteries. For digital biomarkers derived from other single/multiple cognitive tests, they also showed good diagnostic performance with sensitivity and specificity larger than 75%, and AUC larger than 0.7. A composite of score in Trail-Making Test (TMT) and age achieved the best diagnosis performance (sensitivity = 97.0%, specificity = 92.0%, AUC = 0.97) [58]. Moreover, Digital biomarkers retrieved from handwriting/drawing tests showed similar diagnosis performance with computerized test batteries (sensitivity: 74–89.7%, specificity: 70–100%, AUC: 0.84–0.92). Performance on Cross Pentagons Drawing test provided highest diagnostic value (sensitivity = 89.7%, specificity = 100%) [68]. Lastly, digital biomarkers from performing computer-simulated daily living tasks (i.e., SIMBAC) showed an overall satisfactory diagnostic performance, with a sensitivity of 70.0%, specificity of 82.0%, and AUC of 0.84.

As shown in Table 2, when compared with traditional paper-and-pencil tests, there were 13 computerized tests that reported comparable performance with traditional tests in diagnosing MCI, dementia, or CI. Nine computerized tests reported better diagnostic performance in diagnosing MCI, dementia, or CI, and 2 computerized tests reported inferior diagnostic performance than traditional tests in diagnosing MCI, yet still showed acceptable AUC (0.80 and 0.74, respectively). Therefore, most of the computerized tests showed comparable or even better diagnostic performance than traditional paper-and-pencil tests.

### 3.6. Quality of Included Studies

Among the 78 included studies, 71 (91%) of them were reported as having high quality with a total quality score higher than 7. All the computerized memory tests, daily living tasks, and serious games were identified as having high quality. The remaining 7 studies were identified as having high risk because having a quality score range between 5 and 6, with 1 study from other single/multiple cognitive tests, 4 studies from computerized test battery, 1 study from handwriting/drawing tests, and 1 study from daily living tasks and serious games. Among the high-risk studies, 5 studies rated 6 on the NOS, 4 of them did not report matching or using statistical control for age and education level [38,47,57,76], and 1 study did not report educational level [73]. In total, 1 out of these 5 studies did not provide the information on the method of confirming the cognitive status of normal controls, and 4 of them did not adopt the same procedure for ascertainment for control and cases [39,57,73,76]. In addition, another 2 studies rated 5 on the NOS, as they did not report matching or control age and education level and did not use the same procedure for ascertainment for control and cases [77,78]. Additionally, 1 of these 2 studies did not provide the information on how to confirm the cognitive status of normal controls [77], and another one only conducted an interview on the subjective memory complaints and medical history of neurological disease on the control group, without blinding to case/control status [78].

## 4. Discussion

The current review aims to examine the evidence of digital cognitive biomarkers for differentiating MCI and/or dementia from normal healthy adults. It is found that most of the digital cognitive biomarkers derived from computerized tests showed comparable or even better diagnostic performance than traditional paper-and-pencil tests. Therefore, digital cognitive biomarkers can be a useful tool for screening MC and dementia. In addition, the current review examined the sizes of significant between-group mean differences (i.e., MCI vs. control, dementia vs. control, and CI vs. control) in cognitive outcomes measured by computerized tests, as a large group difference in a specific cognitive outcome may imply that this cognitive outcome can be a sensitive digital biomarker that largely differentiates MCI/dementia from normal healthy adults. Moreover, we extended the search of group differences to studies that did not report their diagnostic performance, as the significant group differences in the performances of computerized tests may also provide information on which cognitive outcomes have the potential to become digital cognitive biomarkers in the future. The results showed that digital cognitive biomarkers, especially those related to memory and executive functions, and novel digital biomarkers (e.g., time of pen on surface and in-air, and pen stroke position in handwriting/drawing tests, think time in a serious game) can be sensitive and promising tools for MCI and dementia.

In various cognitive outcomes measured by memory tests and other single/multiple cognitive tests, moderate to large group differences were observed between MCI and controls, dementia and controls, as well as CI and controls. It was mentioned that cognitive functions including memory and executive functions were moderate to largely different between groups, therefore may become the most sensitive digital biomarkers that contribute to the diagnosis or have the potential to become future digital biomarkers. On the other hand, group differences varied from small to large in test batteries. However, the large group differences were consistently observed in learning/memory across different test batteries, suggesting that memory is the most solid and sensitive indicator for MCI, dementia, and CI. Small to moderate group differences were observed in visuospatial skills, orientation, and psychomotor skills measured in test batteries, suggesting that these outcomes may be less sensitive for differentiating MCI and dementia from controls. For handwriting/drawing tests, daily living tasks, and serious games, group differences also varied from small to large. These tests measured more novel outcomes, such as time on the surface and time in the air for the digital Clock Drawing Test [37], average think time in Computer-based Klondike Solitaire [17,18], and stroke position control and pause time per stroke in Chinese Handwriting task [48]. However, it remains unknown why some of these novel outcomes showed large group differences and may become sensitive digital biomarkers, while others showed only small to moderate group differences and may not be sensitive. Future studies can investigate the cognitive functions underlying these novel outcomes. Overall, the group difference in cognitive outcomes measured by computerized tests was larger in the dementia vs. control comparison than MCI vs. control comparison. This result was reasonable, as individuals with dementia have a greater level of cognitive impairments than individuals with MCI, and show a larger deviation from the normal cognition. Moreover, the group difference in CI vs. control comparison was smaller than in dementia vs. control but larger than MCI vs. control comparison, which may be due to the inclusion of different levels of cognitive impairment in the CI group.

For the diagnostic performance of different types of computerized tests, most of the memory tests achieved sensitivity and specificity higher than 70% and acceptable AUC higher than 0.7. This result is consistent with the observation of group differences, suggesting that memory is a sensitive indicator for MCI and dementia. Computerized memory tests (MITSI, CANTAB-PAL) based on paired-associate learning (PAL) test achieved the best diagnostic performance for MCI [52,64], suggesting that PAL can be a good choice when using a single test to screen MCI within a short time. Most of the test batteries achieved even better diagnostic performance, with sensitivity and specificity larger than 75%, and acceptable AUC larger than 0.8. For screening MCI, BoT [66] and MoCA-CC [42] were preferred test batteries. For screening dementia, CST [69], CCS [70], CANS-MCI [71], eSAGE [72], and Computerized Screening Test Battery [73] were preferred. BHA [44,65] was suitable for screening MCI, dementia, and various level of cognitive impairments (CI). The learning/memory subset from CogState was also found to be sensitive in detecting MCI and dementia [43]. Moreover, most of the digital biomarkers from handwriting/drawing tests, daily living tasks, and serious games also provided similar diagnostic performance with test batteries, which encourages the future development of novel tests. Lastly, digital cognitive biomarkers showed better diagnostic performance for dementia and CI than MCI.

Computerized tests have several advantages over paper-and-pencil tests. First, the administration procedures of computerized tests can be more consistent and standardized with less experimenter bias. It also enables precise control of the stimulus presentation time and more accurate measurement. This may explain why most of the computerized tests showed comparable or even better diagnostic performance than traditional paper-and-pencil tests. Secondly, computerized tests are shorter in administration time, more cost-effective, and more accessible than paper-and-pencil tests. Additionally, the administration of computerized tests requires less training on experimenters, as some of them can be administered with the help of caregivers or nurses, or even self-administered at home. Therefore, computerized tests are more suitable for reaching people in remote areas, collecting data from a large sample size (e.g., thousands of participants), and establishing test norms. Lastly, as we mentioned before, computerized tests can collect multiple digital biomarkers simultaneously. It also allows for performing frequent assessments on participants, which can be beneficial for increasing the reliability of measurements, and long-term monitoring of cognitive functions.

Demographical information may also be combined with digital cognitive biomarkers to detect MCI and dementia. It was suggested that the incidence and risk of dementia could vary largely, depending on the ethnicity and race. One study showed a discrepancy as high as 65% between the dementia risk of African Americans and Asian-Americans [79]. Therefore, race/ethnicity may be a useful information in predicting cognitive and dementia outcomes and can be easily collected. However, only a small proportion (20%) of the included studies have provided information on race/ethnicity. Future studies may consider including a diverse race/ethnicity sample, and explore the role of race/ethnicity in predicting MCI and dementia. In addition to the race/ethnicity, family health history may also be another potential biomarker. Rubino and colleagues suggested that compared with older adults without centenarian parents, those with centenarian parents are more resistant to immune aging due to potential genetic and familial lifestyle factors [80], which may further protect them from neurological disorders such as MCI and dementia [81,82]. Therefore, family health history such as the longevity of parents may also be collected in future studies so to provide a better prediction of MCI and dementia.

Around 90% of the studies included in our review were identified as having high quality based on NOS rating. However, eight studies did not match or control for both age and educational level, including one study using the Digital Trail-Making Test-Black and White (dTMT-B&W) [47], Self-Administered Tasks Uncovering Risk of Neurodegeneration (SATURN) [76], BrainCheck [83], Computerized Screening Test System [77], Computer-Based Cognitive Assessment Tool (CompBased-CAT) [61], Cambridge Cognitive Examination administered by Computerized Adaptive Testing (CAMCOG-CAT) [78], one study using digital clock drawing test [57], and a serious game named Episodix [39]. Given that dementia is an age-related disease, and education level was suggested to be associated with as the risk for dementia a higher level of education level may protect people against dementia by increasing “intellectual reserve” [84,85]. Therefore, extra caution should be paid when interpreting the group difference and diagnostic performance in these studies. Moreover, two studies included less than 10 participants in the control group, hence the good diagnostic performance [86] and large group difference [87] may be biased.

The current review is the first systematic review examining the group difference between MCI, dementia, and controls in various cognitive outcomes measured by computerized tests. While a recent systematic review by Chan and colleagues [24] examined the overall diagnostic performance of digital cognitive tests, our review further inspected the details of digital biomarkers that contribute to differentiating or diagnosing MCI, dementia, and CI from normal controls. Therefore, our review provided information not only on which test or test battery to choose, but also on which digital cognitive biomarkers can be more sensitive (e.g., digital biomarkers related to memory and executive functions, and some novel digital biomarkers from handwriting/drawing test, serious games, and daily living tasks) for detecting MCI and dementia. This information may be helpful for establishing shorter yet still sensitive screening tests when administration time is limited and also contribute to the understanding of domains of cognitive impairments in MCI and dementia.

Several directions were suggested for future studies. First of all, our review examined digital cognitive biomarkers for dementia and MCI based on case–control or cross-sectional designs. Future studies may explore the use of digital cognitive biomarkers in long-term follow-ups to monitor the conversion from normal cognition to MCI and dementia. Secondly, future studies may utilize the advantage of digital technologies (i.e., able to collect multiple digital biomarkers at the same time) to measure more novel outcomes during the computerized tests, such as response time per trial, number and accuracy of clicks/taps on the screen, and pause time per click/tap. Since these novel outcomes may be objective measures of cognitive functions, it is worth investigating whether these novel outcomes can be potential digital biomarkers that contribute to a better detection of MCI and dementia. Thirdly, some studies recruited an MCI group consisting of aMCI, while some other studies included other types of MCI (i.e., naMCI). Different compositions of the MCI group resulted in a discrepancy across studies on which digital cognitive biomarker (e.g., performance in a whole test battery vs. performance in a single memory subset) can better discriminate/diagnose MCI from the control group. Therefore, future studies may explore whether digital biomarkers from computerized tests could generate cognitive profiles for different types of MCI, in order to screen out specific types of MCI and provide more precise interventions. In addition, more demographical information such as race/ethnicity and family health histories may be collected and combined with digital cognitive biomarkers to improve the screening and prediction of MCI and dementia. Lastly, as we read through the selected studies, only a few of them adopted neuroimaging techniques during the computerized tests. Future studies may use neuroimaging techniques to monitor brain activities during the computerized tests, so to investigate the connection between brain function and behavioral manifestations, and further provide interventions targeting the relevant brain areas.

The current review has several limitations. Firstly, we did not include digital biomarkers derived from passive monitoring, which is an important domain of early detection of MCI and dementia. Secondly, the current review did not consider the comorbidity with mental disorders such as depression and anxiety, which are also important factors that influence cognitive functions.

## 5. Conclusions

Digital cognitive biomarkers from the computerized test can be sensitive and promising tools for differentiating MCI and dementia from healthy normal adults. The current review suggested that digital biomarkers related to memory and executive functions may be more sensitive than digital biomarkers related to other cognitive domains. Future studies may utilize this information to selectively choose digital cognitive biomarkers and computerized tests, so to build time-efficient and sensitive screening tools for MCI and dementia. In addition, novel digital biomarkers from handwriting/drawing tests, daily living tasks, and serious games may also be considered as objective and implicit measures of cognition. In future studies, computerized tests may collect more novel digital biomarkers, so as to provide a better detection of MCI and dementia. Further longitudinal follow ups can be made to explore the potential of digital cognitive biomarkers in predicting the conversion from normal cognition to MCI and dementia, as well as studies combining digital cognitive biomarkers with demographical information (e.g., race/ethnicity, family health history) to collectively predict MCI and dementia.

## Figures and Tables

**Table 1:** Summary on the effect sizes of groups difference in various outcomes of computerized tests.

	**MCI vs. Control**		Dementia vs. Control		CI vs. Control	
	***g*** **Range**	**Most Sensitive Digital Biomarkers**	*g* Range	Most Sensitive Digital Biomarkers	*g* Range	Most Sensitive Digital Biomarkers
**Memory test**	0.7–1.6	MITSI-L (PAL correct pairs A2 and B2); Associative memory tasks (word-word) accuracy; CANTAB-PAL total erros adjusted	2.5–4.9	CANTAB-PAL total errors adjusted The Placing Test	-	-
**Test battery**	0.1–2.9	CANTAB (paired associate learning, rapid visual processing, spatial recognition memory); MoCA-CC (total score, delayed recall, attention); CogState (learning/working memory composite) total score	0.3–7.2	CANTAB (paired association learning); NeuroTrax Mindstreams (verbal memory accuracy); Inbrain CST (memory score)	0.2–2.1	BHA (“Favorite (memory)” total correct); CDST (“memory impairment screen” score; “spatial span test” total span); CoCoSc total score; CompBased-CAT total z-score; BoCA (attention score, total score)
**Handwriting/** **drawing tasks**	0.1–2.1	dCDT (time on surface, time in air, total time); Chinese handwriting task (stroke position control, stroke length, stroke orientation, ratio of in air to on paper trajectory length)	0.2–2.4	Chinese Handwriting task (stroke position control, pause time per stroke, stroke orientation); dCDT (total completion time, time in air, minute hand distance from center in command trial, hour hand distance from center in copy trial)	-	-
**Daily living task and Serious game**	0.1–1.3	Computer-based Klondike Solitaire (average think time, average accuracy, total time SD); SIMBAC (total completion time); Computerized Touch-Panel Games (“flipping cards” completion time)	0.8–2.9	SIMBAC (total accuracy, total completion time); Computerized Touch-Panel Games (“arranging pictures (processing and remote memory)” completion time; “beating devils (judgment)” accuracy; “flipping cards (recent memory)” completion time; “finding mistakes (attention and discrimination)” completion time)	0.7–1.2	CFSAT accuracy (“Ugreens website”, “Internet banking”, “medication management”, “ATM task”, “Ticket task”)
**Other single/multiple cognitive tests**	0.7–1.2	correct cancellations in e-CT; inspection Time (IT) test score; dTMT-B&W-A completion time	0.9–3.1	Computerized SRT task and FRT score adjusted for age and education; Correct cancellation in e-CT	1.0–2.0	TMT (total score, total completion time, total response time)

Abbreviations. BHA: Brain Health Assessment; BoCA: Boston Cognitive Assessment; CANTAB: Cambridge Neuropsychological Test Automated Battery; CDST: Computerized Dementia Screening Test; CFSAT: Computer-based functional skills assessment and training; CoCoSc: Computerized Cognitive Screen; CompBased-CAT: CompBased administered by Computerized Adaptive Testing; dCDT: digital Clock Drawing Test; dTMT-B&W-A: digital Trail-Making Test—Black and White—Part A; e-CT: electronic version of Cancellation Test; FRT: Flanker Reaction Time; Inbrain CST: Inbrain Cognitive Screening Test; MITSI-L: The Miami Test of Semantic Interference; PAL: Paired Associate Learning; SIMBAC: SIMulation-Based Assessment of Cognition; SRT: Simple Reaction Time; TMT: Trail-Making Test; MoCA-CC: Computerized Tool for Beijing version of The Montreal Cognitive Assessment (MoCA).

**Table 2:** Summary on diagnostic performance of computerized tests and the comparison paper-and-pencil tests.

	Sen (%)	Spec (%)	AUC	Computerized Tests vs. Paper-and-Pencil Tests	Whether Computerized Test Is Better
**MCI**					
***Memory test***	42.0–85.8	66.0–93.3	0.53–0.93	CANTAB-PAL vs. CERAD wordlist learning delay recall	inferior
				MemTrax vs. MoCA-BJ	better
				Digital VSM vs. Cube-copying test	much better
				digital TPT vs. paper-and-pencil TPT	better
***Test battery***	41.4–100.0	64.0–100.0	0.65–0.97	CANS-MCI vs. MoCA, ACE-R	comparable
				subsets in NeuroTrax MindStreams vs. subsets in WMS-III, RAVLT, CDT, TMT-A, Boston Naming Test, COWA	comparable and some subsets are even better
				memory factor in Tablet-based cognitive assessments vs. MMSE	inferior
				BHA vs. MoCA	better
				CAMCI vs. MMSE	better
				COMCOG-CAT vs. CAMCOG	comparable
***Handwriting/drawing test***	71.4–100.0	56.0–100.0	0.77–0.89	machine learning on dCDT features vs. CERAD	comparable
***Daily living task and Serious game***	76.9–84.4	58.0–88.9	0.77–0.90	SASG vs. MoCA	comparable
				SIMBAC vs. MMSE, Composite score of RAVLT-Delayed recall, Boston Naming Test, Digit Span, Digit Symbol Coding, and TMT-B	comparable
***Other single/multiple cognitive test***	56.3–84.7	53.6–90.5	0.67–0.91	e-CT vs. K-T CT	comparable
**Dementia**					
***Memory test***	88.9	92.9	-	digital TPT vs. paper-and-pencil TPT	comparable
***Test battery***	52.9–100.0	56.0–100.0	0.54–0.99	CST vs. MMSE	better
				CCS vs. MoCA	inferior
				BHA vs. MoCA	comparable
***Handwriting/drawing test***	82.0–97.7	71.4–86.0	0.90–0.92	dCDT parameters vs. CERAD	comparable
***Daily living task and Serious game***	86.0	75.0	0.97	SIMBAC vs. MMSE, Composite score of RAVLT-Delayed recall, Boston Naming Test, Digit Span, Digit Symbol Coding, and TMT-B	comparable
***Other single/multiple cognitive tests***	62.7–86.1	75.0–95.3	0.76–0.95	e-CT vs. K-T CT	comparable
**CI**					
***Memory test***	91.8	72.0	0.89	-	-
***Test battery***	70.7–91.0	69.0–94.2	0.78–0.95	BHA vs. MoCA	better
				eSAGE vs. paper version of SAGE	better
***Handwriting/drawing test***	74.0–89.7	70.0–100.0	0.84–0.92	machine learning on dCDT vs. MMSE	better
***Daily living task and Serious game***	70.0	82.0	0.84	-	-
***Other single/multiple cognitive tests***	77.0–97.0	80.6–92.6	0.77–0.97	TMT vs. MMSE	comparable
				e-CT vs. K-T CT	comparable

Abbreviations. ACE-R: Addenbrooke’s Cognitive Examination-Revised; BHA: Brain Health Assessment; CANTAB: Cambridge Neuropsychological Test Automated Battery; CAMCI: Computer Assessment of Memory and Cognitive Impairment; CDT: Clock Drawing Test; CERAD: The Consortium to Establish a Registry for Alzheimer’s Disease; COMCOG: Computer-assisted Cognitive Rehabilitation; COMCOG-CAT: Computer-assisted Cognitive Rehabilitation administered by Computerized Adaptive Testing; COWA: Controlled Oral Word Association Test; e-CT: electronic version of Cancellation Test; e-SAGE: electronic version of Self-Administered Gerocognitive Examination; MMSE: Mini-Mental State Examination; RAVLT: Rey Auditory Verbal Learning Test; PAL: Paired Associate Learning; SAGE: Self-Administered Gerocognitive Examination; SASG: Smart Aging Smart Game; SIMBAC: SIMulation-Based Assessment of Cognition; TMT-A: Trail-Making Test—Part A; TMT-B: Trail-Making Test—Part B; MoCA: The Montreal Cognitive Assessment (MoCA); MoCA-BJ: Beijing version of The Montreal Cognitive Assessment (MoCA); VSM: Visuo-spatial Memory task; WMS-III: Wechsler Memory Scale, 3rd edition.

## Data Availability

The data that support the findings of this study are available from the corresponding author upon reasonable request.

## Supplementary Materials

The following supporting information can be downloaded at: https://www.mdpi.com/article/10.3390/jcm11144191/s1, Table S1: Demographical characteristics of the included studies. Table S2: Characteristics of computerized tests. Table S3: Diagnostic performance of digital cognitive biomarkers for mild cognitive impairments (MCI). Table S4: Diagnostic performance of digital cognitive biomarkers for dementia. Table S5: Diagnostic performance of digital cognitive biomarkers for cognitive impairments (CI). Table S6: Quality assessment based on The Newcastle–Ottawa Scale (NOS) [88,89,90,91,92,93,94,95,96,97,98,99,100,101,102,103,104,105,106,107,108,109,110,111,112,113,114,115,116,117]. Figure S1: Between-group mean difference in cognitive outcomes measured by computerized tests in mild cognitive impairments (MCI) and normal controls. Figure S2: Between-group mean difference in cognitive outcomes measured by computerized tests in dementia and normal controls. Figure S3: Between-group mean difference in cognitive outcomes measured by computerized tests in cognitive impairments (CI) and normal controls.
