# Supplementary material for: Digital Cognitive Biomarker for Mild Cognitive Impairments and Dementia: A Systematic Review
Source: J Clin Med. 2022 Jul 19;11(14):4191. doi: 10.3390/jcm11144191 (PMC9320101; doi:10.3390/jcm11144191)
Supplement: Supplementary file 1 [file jcm-11-04191-s001.zip › Table S1_demographics.pdf]

Table S1. Demographical characteristics of the control group, the mild cognitive impairment (MCI) group, and the cognitively impaired (CI) group of the included studies.

			Control			MCI			Dementia			CI		
**Author**	**Year**	**Race/ ethnicity**	**n (female)**	**Age (SD)**	**Education years (SD)**	**n (female)**	**Age (SD)**	**Education years (SD)**	**n (female)**	**Age (SD)**	**Education years (SD)**	**n (female)**	**Age (SD)**	**Education years (SD)**
**Memory test**														
Alegret et al. [88]	2020	-	154 (93)	67.98 (7.92)	12.62 (4.18)	122 (80)	66.86 (8.34)	-	-	-	-	-	-	-
Curiel et al. [64]	2016	-	64 (53)	74.0 (7.3)	15.2 (2.9)	34 (15)	77.6 (6.3)	14.7 (3.8)						
Junkkila et al. [52]	2012	-	22 (14)	70 (4.48)	10 (3.25	17 (7)	73 (6.3)	8 (3)	19 (14)	73 (6.76)	8 (2.88)	-	-	-
Liu et al. [89]	2021	-	50 (20)	68 (8.19)	median = 9 (IQR = (4, 12))	50 (26)	67.7 (10.79)	median = 9 (IQR = (6, 12))	-	-	-	-	-	-
Maki et al. [16]	2010	-	29 (-)	78.3 (5.3)	-	10 (-)	73.7 (10.3)	-	-	-	-	-	-	-
Rafii et al. [74]	2011	-	25 (14)	80.3 (8.6)	16.3 (2.4)	12 (4)	74.8 (9.0)	14.7 (2.5)	49 (18)	76.4 (9.8)	15.2 (2.8)	-	-	-
Ramratan et al. [90]	2012	Control: 71.6% Caucasian; aMCI: 60.9% Caucasian,	303 (114)	78.5 (5.2)	14.3 (3.4)	87 (41)	81.5 (5.5)	13.1 (3.4)	-	-	-	-	-	-
Troyer et al. [91]	2016	-	21 (12)	72.9 (6.7)	14.3 (2.6)	24 (15)	76.1 (7.6)	14.2 (2.5)	-	-	-	-	-	-
Vacante et al. [53]	2013	-	40 (21)	75.73 (7.45)	16.1 (3.24)	20 (8)	79 (7.17)	15.45 (2.90)	18 (5)	75.45 (5.78)	15.55 (2.97)			
van der Hoek et al. [92]	2019	-	45 (27)	82.6 (4.9)	11.0 (range: 8.0 - 14.0)	37 (28)	84.7 (5.4)	10.0 (range: 8.0 - 12.0)				-	-	-
**Test battery**														
Ahmed et al. [93]	2012	-	20 (9)	77.4 (4)	14.7 (2.9)	15 (10)	80.9 (7.2)	13.1 (3)	-	-	-	-	-	-
Bissig et al. [76]	2020	93% non-Hispanic Caucasian	37 (22)	65.5 (9.2)	16.3 (2.5)	-	-	-	24 (11)	71.5 (10.3)	-	-	-	-
Cerino et al. [94]	2021	45% non-Hispanic white, 40% non-Hispanic Black, 10% Hispanic White, 3% Hispanic Black, 1%	211 (142)	77.06 (4.8)	15.35 (3.46)	100 (67)	78.3 (4.9)	14.2 (3.66)	-	-	-	-	-	-

		Asian, other < 1%, more than one race < 1%												
Chan et al. [95]	2020	-	75 (60)	70.2 (8.1)	primary level or below (29), secondary level or above (46)	37 (19)	76.1 (6.3)	primary level or below (18), secondary level or above (19)	33 (26)	78.6 (7.1)	primary level or below (18), secondary level or above (15)	-	-	-
Chin et al. [15]	2020	-	26 (23)	68.46 (6.28)	12.62 (6.28)	42 (24)	71.69 (7.3)	12.57 (3.89)	29 (-)	73.62 (8.74)	12.17 (4.48)	-	-	-
Cho et al. [59]	2002	-	103 (55)	62.73 (5.85)	10.58 (5.13)	-	-	-	-	-	-	41 (23)	69.22 (6.72)	8.17 (5.77)
Cho et al. [87]	2008	-	8 (5)	65.3 (6.9)	14 (2.1)	48 (28)	71.8 (8.6)	12.1 (2.8)				-	-	-
Darby et al. [96]	2002	100% Caucasian	40 (-)	-	-	20 (-)	-	-	-	-	-	-	-	-
Dorociak et al. [97]	2021	88% White	44 (11)	72.4 (5.2)	15.1 (2.4)	25 (4)	74 (6)	15.2 (2.6)	-	-	-	-	-	-
Dougherty et al. [69]	2010	-	104 (63)	75.45 (7.25)	15 (2.7)	27 (3)	67.24 (6.42)	14 (4.07)	84 (49)	76.69 (7.31)	13.77 (2.91)	-	-	-
Dwolatzky et al. [55]	2003	-	39 (26)	73.41 (8)	14.95 (3.5)	30 (13)	77.15 (6.43)	13.07 (2.86)	29 (16)	80.55 (4.91)	11.31 (2.85)	-	-	-
Égerházi et al. [41]	2007	-	3000 (-)	-	-	25 (-)	55 (6)	-	15 (-)	58 (6)	-	-	-	-
Fichman et al. [98]	2008	-	97 (-)	69.46 (6.19)	6.30 (4.91)	-	-	-	47 (-)	72.03 (5.60)	9.15 (5.15)	-	-	-
Green et al. [99]	1994	-	50 (-)	68.7 (5)	15.4 (2.3)	-	-	-	52 (-)	71.2 (10.1)	14.7 (2.3)	-	-	-
Groppell et al. [83]	2019	-	65 (55)	62.9 (16.5)	-	-	-	-	19 (17)	75 (9.5)	-	-	-	-
Gualtieri & Johnson [100]	2005	-	89 (-)	63.13 (-)	-	36 (-)	66.11 (-)	-	53 (-)	62.23 (-)	-	-	-	-
Huang et al. [54]	2019	-	41 (24)	median = 75 (IQR = 12.5)	median = 6.0 (IQR = 3.0)	43 (28)	median = 75 (IQR = 10.0)	median = 7.0 (IQR = 9.0)	36 (26)	median = 80.0 (IQR = 11.8)	median = 6.0 (IQR = 7.8)	-	-	-

Inoue et al. [77]	2005	-	55 (44)	72.6 (7.3)	-	22 (14)	72 (9.6)	-	29 (21)	78.1 (5.2)	-	-	-	-
Inoue et al. [73]	2009	-	102 (65)	77.1 (5.8)	-				72 (68)	80.0 (5.1)	-	-	-	-
Maruff et al. [43]	2013	-	659 (381)	69.5 (6.6)	12 (range: 9-15)	107 (54)	75.7 (7.5)	12 (range: 9-15)	44 (26)	79.3 (7.2)	12 (range: 9-15)	-	-	-
Memória et al. [71]	2014	-	41 (33)	71.68 (4.62)	13.41 (4.45)	35 (27)	73.8 (5.5)	11.25 (4.08)	21 (9)	76.14 (4.98)	11.57 (4.85)	-	-	-
Mundt et al. [101]	2007	94% White, 3% Asian, 1% Black, 2% others	36 (-)	75.6 (5.3)	16.4 (2.2)	37 (-)	75.9 (6.1)	16.2 (2.5)	34 (-)	78.5 (4.9)	15.2 (2.9)	-	-	-
Possin et al. [65]	2018	-	137 (78)	75.6 (6.3)	17.4 (2.1)	71 (22)	70.24 (9.36)	16.72 (2.91)	30 (14)	69.1 (9.9)	17 (2)	-	-	-
Rodríguez-Salgado [44]	2021	-	53 (39)	70.4 (5.9)	16.2 (4.1)	46 (24)	72.7 (7.5)	14.2 (4.1)	47 (33)	74.1 (5.9)	13 (5.2)	-	-	-
Ruano et al. [66]	2019	-	267 (whole control sample) (133)	57.4 (11.4)	13.6 (4.5)	-	-	-	-	-	-	17 (11)	70.2 (8)	5.3 (1.9)
Ruano et al. [102]	2016	-	39 (-)	72.2 (7.2)	4.1 (2.5)	-	-	-	-	-	-	39 (-)	73 (7.5)	4.2 (2.4)
Saxton et al. [35]	2009	94.3% White	296 (199)	71.84 (5.95)	13.74 (2.69)	228 (142)	75.18 (6.76)	13.1 (2.61)	-	-	-	-	-	-
Scanlon et al. [70]	2016	-	20 (8)	72.5 (12)	completed primary (2), attended secondary (7), completed secondary (5), attended tertiary (6)	-	-	-	40 (23)	76.5 (12.5)	completed primary (12), attended secondary (20), completed secondary (10), attended tertiary (10)	-	-	-
Scharre et al. [72]	2017	89% Caucasian	21 (44)	75.2 (7.3)	15.1 (2.7)	24 (-)	-	-	21 (-)	-	-	-	-	-

Takahashi et al. [61]	2021	-	746 (-)	-	-	-	-	-	-	-	-	27 (-)	-	-
Veroff et al. [103]	1991	-	33 (23)	74.8 (5.1)	12 (3)	-	-	-	15 (5)	78.5 (5.9)	14 (4)			
Vyshedskiy et al. [62]	2022	-	50 (36)	70.6 (13.1)	16.2 (2.3)	-	-	-	-	-	-	50 (32)	70 (1.42)	16.2 (16.1)
Wong et al. [60]	2017	-	101 (79)	70.5 (86)	low education (<=6 years) (30), high education (> 6 years) (71)	-	-	-	-	-	-	59 (45)	78.2 (8.1)	low education (<=6 years) (45), high education (14)
Wouters et al. [78]	2009	-	41 (30)	median: 79.7 (range: 61.5 - 90.4)	4.8 (1.3)	21 (12)	median: 82.1 (range: 69.8 - 94.1)	4.8 (0.9)	22 (18)	median: 82.5 (range: 74.5 - 88.5)	4.4 (1.1)	-	-	-
Ye et al. [104]	2022	-	35 (25)	67.8 (9.6)	Some college or less (2), Bachelor (10), Post-bachelor's degree (14), N/A (9)	22 (8)	73.5 (5.9)	Some college or less (2), Bachelor (6), Post-bachelor's degree (9), N/A (5)	42 (16)	71.5 (9)	Some college or less (8), Bachelor (11), Post-bachelor's degree (16), N/A (16)	-	-	-
Yu et al. [42]	2015	-	55 (13)	72,2 (5.0)	15.8 (3.7)	63 (18)	73.6 (5.1)	14.7 (3.6)				-	-	-
Zhang et al. [105]	2017	-	42 (29)	72.64 (9.08)	14.19 (3.61)	-	-	-	-	-	-	65 (28)	77.26 (8.37)	10.71 (4.68)
**Other single/multiple cognitive tests**														
Angelillo et al. [106]	2019	-	29 (-)	65 (13)	>= 5 years (-)	-	-	-	36 (-)	75 (9)	>= 5 years (-)	-	-	-
Bonney et al. [46]	2006	-	28 (16)	74.5 (7)	11.5 (2.5)	28 (16)	74 (7.4)	11.4 (2.9)	-	-	-	-	-	-
Cheah et al. [67]	2022	100% Asian	59 (33)	62.58 (5.89)	15.05 (2.82)	59 (31)	67.51 (6.3)	13.12 (3.2)	-	-	-	-	-	-
			30 (19)	73.4 (7.24)	15.03 (2.66)	-	-	-	30 (20)	77.67 (6.96)	21.33 (2.8)	-	-	-
Chen et al. [56]	2017	-	28 (16)	73.7 (5.4)	7.7 (5)	33 (23)	74.9 (5.6)	5.6 (4)	26 (16)	79.5 (6.1)	4.9 (3.9)	-	-	-

García-Casal et al. [107]	2019	-	69 (26)	73.14 (6.29)	7.76 (3.45)	59 (27)	77.6 (5.01)	7.09 (3.89)	84 (32)	78.27 (5.81)	6.86 (2.83)	-	-	-
Kalová et al. [108]	2005	-	10 (8)	60.2 (2.9)	13.9 (0.8)	-	-	-	11 (5)	69.2 (1.4)	14.2 (0.9)	-	-	-
Kokubo et al. [58]	2018	-	29 (18)	55.8 (13.7)	15.5 (3.8)							27 (11)	49.4 (7.6)	13.8 (2.3)
Lunardini et al. [109]	2020	-	22 (16)	76.2 (4.2)	12.4 (4.4)	49 (23)	78 (5.4)	10.3 (4.6)	12 (8)	78.6 (4)	8.8 (5.1)	-	-	-
Mollica et al. [110]	2017	-	47 (-)	65.4 (6.6)	11.5 (4.5)	-	-	-	15 (-)	68.1 (8.9)	9.9 (4.6)	-	-	-
Simfukwe et al. [47]	2021	-	22 (14)	53 (1.48)	12 (2.48)	22 (15)	67.19 (6.03)	12.52 (3.72)	-	-	-	-	-	-
Wu et al. [45]	2017	-	112 (84)	74.7 (6.94)	education > 12 years (79), education <= 12 years (33) (-)	129 (81)	76.5 (7.49)	education > 12 years (48), education <= 12 years (81)	84 (48)	76.5 (5.94)	education > 12 years (45), education <= 12 years (39)	-	-	-
Zhou et al. [86]	2017	-	11 (6)	80.5 (6.3)	15.2 (3)	8 (5)	85.2 (4.5)	13.8 (2.3)	9 (2)	80.8 (6.6)	14.6 (1.8)	-	-	-
**Handwriting/drawing test**														
Amini et al. [111]	2021	NC: 90.6% White, 2.7% Black, 2.6% Asian, 2.5 Hispanic, 1.6% other; Dementia: 97.5% White, 1.2% Hispanic, 0.6% Black, 0.6% Asian	3263 (1773)	61.8 (13.2)	attending high school (45), graduating from high school (574), attending college (761), graduating from college (1831)	-	-		160 (86)	82.1 (7.3)	12 attending high school, 61 graduating from high school, 42 attending college, 45 graduating from college (-)	-	-	-

Binaco et al. [112]	2020	99% Caucasian	35 (18)	77.1 (6.66)	14.46 (2.85)	69 (44)	75.6194 (7.2998)	13.6239 (2.9756)	59 (30)	79.71 (5.34)	-	-	-	-
Davoudi et al. [57]	2020	96.54% White	175 (80)	68.37 (5.76)	16.39 (2.54)	-	-	-	56 (38)	80.04 (6.28)	12.75 (3.07)	-	-	-
Garre-Olmo et al. [68]	2017	-	17 (-)	70.2 (7.4)	-	12 (-)	63.5 (6.5)	-	23 (-)	72.6 (7.9)	-	-	-	-
Ishikawa et al. [113]	2019	-	36 (21)	70 (5)	-	25 (15)	75.9 (5.3)	-	10 (7)	76.7 (6)	-	-	-	-
Matusz et al. [114]	2022	100% White	21 (-)	76.1 (5.82)	15.2 (2.93)	21 (-)	73..9 (6.67)	13.8 (2.16)	-	-	-	-	-	-
Müller, et al. [37]	2019	-	137 (60)	69.6 (7.8)	13.2 (2.7)	138 (66)	70.8 (8.4)	12.6 (2.9)	106 (55)	71.4 (8.4)	12.6 (2.6)	-	-	-
Robens et al. [49]	2019	-	67 (25)	65.9 (10.3)	14.1 (3)	64 (35)	67.9 (11.2)	11.9 (2.9)	56 (40)	72.7 (9.2)	11.1 (2.9)	-	-	-
Souillard-Mandar et al. [115]	2021	Training set: 95.8% White/Caucasian, 2.3% Asian, 1.9% Black/African American; Testing set: 95.9% White/Caucasian, 2.3% Asian, 1.8% Black/African American	512 (449)	69.88 (8.32)	college graduate (476), some college (245), HS graduate (167), Less than HS (33)	-	-	-	-	-	-	409 (-)	-	-
Yu & Chang [48]	2019	-	18 (5)	75.8 (5.8)	8.9 (3.6)	14 (4)	74.9 (5.2)	9.1 (2.9)	22 (7)	74.6 (4.9)	9.6 (3.2)	-	-	-
**Daily living task & Serious game**														
Cabinio et al. [116]	2020	-	107 (45)	76.47 (3.03)	10.95 (4.09)	32 (17)	76.75 (5.3)	10.75 (3.84)	-	-	-	-	-	-
Fukui et al. [50]	2015	-	75 (49)	75.1 (6.1)	11.9 (2.3)	41 (25)	75.3 (6.5)	12.3 (2.1)	124 (83)	75.6 (5.9)	11.3 (2.3)	-	-	-
Gielis et al. [17,18]	2021a & b	-	23 (11)	70 (5.4)	primary to lower secondary	23 (13)	80 (5.2)	primary to lower secondary	-	-	-	-	-	-

					education (5), upper secondary to post-secondary non-tertiary education (7), short-cycle tertiary education to Bachelor's or equivalent level (11)			education (4), upper secondary to post-secondary non-tertiary education (13), short-cycle tertiary education to Bachelor's or equivalent level (6)						
Harvey et al. [63]	2021	Control: 31% Hispanic, 31% non-Hispanic White, 33% non-Hispanic Black, 5% Asian; CI: 15% Hispanic, 10% non-Hispanic White, 73% non-Hispanic Black, 2% Asian	62 (54)	73.19 (6.39)	15.67 (2.41)	-	-	-	-	-	-	55 (39)	75.15 (6.39)	13.93 (2.73)
Isernia et al. [19]	2021	-	74 (37)	75.47 (2.66)	5-8 years (29), 9-13 years (34), more than 13 years (11)	61 (34)	74.20 (5.02)	5-8 years (29), 9-13 years (21), more than 13 years (10)				-	-	-

Rapp et al. [51]	2018	90% White	81 (52)	70.5 (7.8)	higher than high school (77), lower or at high school (4)	52 (31)	75.6 (6.8)	higher than high school (41), lower or at high school level (11)	28 (16)	77.5 (7.1)	education level > high school (14), education level <= high school (14)	-	-	-
Valladares-Rodriguez et al. [39]	2018b	-	28 (12)	75.57 (7.14)	1.34 (0.82)	16 (6)	76.87 (9.33)	1.78 (0.41)	20 (12)	79.15 (4.91)	1.8 (1.19)	-	-	-
Vallejo et al. [117]	2017	-	20 (8)	74.5 (5.9)	12.1 (3.4)	-	-	-	18 (9)	77.8 (6.2)	11.6 (2.6)	-	-	-
