# Supplementary material for: Digital Cognitive Biomarker for Mild Cognitive Impairments and Dementia: A Systematic Review
Source: J Clin Med. 2022 Jul 19;11(14):4191. doi: 10.3390/jcm11144191 (PMC9320101; doi:10.3390/jcm11144191)
Supplement: Supplementary file 1 [file jcm-11-04191-s001.zip › Table S2_computerized test characteristics.pdf]

Table S2. Characteristics of the computerized tests.

Author	Year	Test Name	Administration time	Device	Supervision Level
Memory test					
Alegret et al. [88]	2020	FACEmemory	30 min	tablet with voice recognition and touchscreen	minimal supervision
Curiel et al. [64]	2016	The Miami Test of Semantic Interference (MITSI-L)	8-10 min	touch-screen computer	unsupervised
Junkkila et al. [52]	2012	CANTAB-PAL Computerized Memory Test	15 min	touch-screen computer	supervised
Liu et al. [89]	2021	MemTrax memory test	2-5 min	online APP using digital devices with touch screen	supervised
Maki et al. [16]	2010	Computerized Visuospatial Memory Test (VSM)	-	computer with a touch panel screen	supervised
Rafii et al. [74]	2011	Mild Cognitive Impairment Screen (MCIS) (digital version of CERAD Word List Memory (WLM) test)	10 min	computer	supervised
Ramratan et al. [90]	2012	Computerized Cued-Recall Retrieval Speed Test (CRRST)	-	computer and microphone	
Troyer et al. [91]	2016	Computer-based Associative Memory Tasks	-	computer	-
Vacante et al. [53]	2013	Computerized versions of The Placing Test	-	computer with mouse	-
van der Hoek et al. [92]	2019	Computerized memory test (MemTrax Test)	< 5 min	computer	supervised
Test battery					
Ahmed et al. [93]	2012	Computer-Administered Neuropsychological Screen for Mild Cognitive Impairment (CANS-MCI)	30 min	computer	minimal supervision
Bissig et al. [76]	2020	Self-Administered Tasks Uncovering Risk of Neurodegeneration (SATURN)	12 min	tablet	-
Cerino et al. [94]	2021	Smartphone-based digital Ecological Momentary Assessments (EMA)	20-25 min per day for 16 consecutive days	smart phone	unsupervised
Chan et al. [95]	2020	Electronic Cognitive Screen Technology (EC-screen)	-	tablet	unsupervised or minimal supervision
Chin et al. [15]	2020	Inbrain Cognitive Screening Test (Inbrain CST)	-	tablet	supervised
Cho et al. [59]	2002	Computerized Dementia Screening Test (CDST)	< 20 min	computer	supervised
Cho et al. [87]	2008	Mild Cognitive Impairment Screen (MCIS) (digital version of CERAD)	10 min	computer	supervised
Darby et al. [96]	2002	CogState	15-18 min, administered four times within 3 hours	computer	supervised
Dorociak et al. [97]	2021	Survey for Memory, Attention, and Reaction Time (SMART)	-	web-based, using computer,	unsupervised

				tablet, or other internet-connected device	
Dougherty et al. [69]	2010	Computer Self-Test (CST)	-	internet-based, using computer	-
Dwolatzky et al. [55]	2003	NeuroTrax Mindstreams computerized cognitive assessment system	45 min	computer with mouse or number pad on the keyboard	supervised
Égerházi et al. [41]	2007	Cambridge Neuropsychological Automated Test Battery (CANTAB)	-	computer	-
Fichman et al. [98]	2008	Computerized Cognitive Screening Battery (CompCogs)	15 min	computer and keypad	
Green et al. [99]	1994	Assessment of Cognitive Skills (ASC)	-	computer	-
Groppell et al. [83]	2019	BrainCheck Memory	-	iPads or iPhones	supervised
Gualtieri & Johnson [100]	2005	Computerized Neurocognitive Screening Battery (CNS Vital Signs)	30 min	computer	minimal supervision
Huang et al. [54]	2019	Tablet-based Cognitive Assessments	10 min	tablet with touch screen	-
Inoue et al. [77]	2005	Computerized Screening Test System	5 min	touchscreen computer	supervised
Inoue et al. [73]	2009	Computerized Screening Test Battery	4 min	touchscreen computer	-
Maruff et al. [43]	2013	CogState Brief Battery	10-15 min	computer	unsupervised
Memória et al. [71]	2014	Brazilian version of Computer-Administered Neuropsychological Screen for Mild Cognitive Impairment (CANSMCI): adaptation of the color-word Stroop task, reaction time subsets with minimal cognitive complexity; immediate and delay recognition; picture naming test	50 minutes	computer	supervised
Mundt et al. [101]	2007	Telephonic Remote Evaluation of Neuropsychological Deficits (TREND)	22 min	telephone	unsupervised
Possin et al. [65]	2018	Brain Health Assessment (BHA)	10 min	tablet	supervised
Rodríguez-Salgado [44]	2021	Brain Health Assessment (BHA)	10 min	tablet	supervised
Ruano et al. [66]	2019	Brain on Track Self-applied Computerized Test (BoT)	24 min	computer	supervised at a training session but unsupervised afterwards
Ruano et al. [102]	2016	Brain on Track self-applied computerized test (BoT)	< 18 min	computer	supervised at a training session but unsupervised afterwards
Saxton et al. [35]	2009	Computer Assessment of Mild Cognitive Impairment (CAMCI)	20 min	computer	minimal

					supervision
Scanlon et al. [70]	2016	Computerized Cognitive Screening (CCS)	-	tablet	minimal supervision
Scharre et al. [72]	2017	digital format of Self-Administered Gerocognitive Examination (eSAGE)	-	tablet	minimal supervision
Takahashi et al. [61]	2021	Computer-Based Cognitive Assessment Tool (CompBased-CAT)	-	tablet or computer	-
Veroff et al. [103]	1991	Computerized Neuropsychological Test Battery (CNTB)	50 min	computer	supervised
Vyshedskiy et al. [62]	2022	Boston Cognitive Assessment (BOCA)	10 min	smartphone or computer	unsupervised
Wong et al. [60]	2017	Computerized Cognitive Screen (CoCoSc)	15 min	touch-screen computer	supervised
Wouters et al. [78]	2009	Cambridge Cognitive Examination administered by Computerized Adaptive Testing (CAMCOG-CAT), CAMCOG-Plus-CAT	20 min	computer	supervised
Ye et al. [104]	2022	BrainCheck	within 50 min	iPads or touchscreen computer	
Yu et al. [42]	2015	Computerized tool for Beijing version of MoCA (MoCA-CC)	10 min	computer	supervised
Zhang et al. [105]	2017	Fun Cube (FC) based Brain Gym (BG) Cognitive Function Assessment System	-	touch-screen computer and fun cubes with wireless sensors	supervised
Other single/multiple cognitive tests					
Angelillo et al. [106]	2019	Digital Attentional Matrices Test (AMT)	-	tablet	supervised
Bonney et al. [46]	2006	Domputerized Visual Inspection Time (IT) task	-	computer	-
Cheah et al. [67]	2022	Digital Rey–Osterrieth Complex Figure	-	tablet and electronic pen	-
Chen et al. [56]	2017	Computerized simple reaction time (SRT) task and flanker reaction time (FRT) task	-	computer	-
Garcia-Casal et al. [107]	2019	Affect-GRADIOR	-	computer	-
Kalová et al. [108]	2005	Computerized Hidden Goal Task (HGT) and Memory Test (MT)	-	computer with mouse	-
Kokubo et al. [58]	2018	User eXperience-Trail Making Test (UX-TMT)	7-13 min	touchscreen tablet	supervised
Lunardini et al. [109]	2020	Digital Trail Making Test Part A and Part B, and digital Bells Test	-	web app on tablet	supervised by a virtual assistant
Mollica et al. [110]	2017	Computer-based Visuomotor Coordination Task	-	computer	supervised
Simfukwe et al. [47]	2021	Digital Trail Making Test-Black and White (dTMT-B&W)	-	web-based app on smartphones and tablets	minimal supervision
Wu et al. [45]	2017	Tablet-based Cancellation Test (e-CT)	-	tablet	supervised
Zhou et al. [86]	2017	Instrumented Trail Making Test (iTMT)	-	computer and wearable sensors	supervised
Handwriting/drawing test					

Amini et al. [111]	2021	Digital Clock Drawing Test (dCDT)	-	electronic pen	supervised
Binaco et al. [112]	2020	Digital Clock Drawing Test (dCDT)	-	electronic pen	-
Davoudi et al. [57]	2020	Digital Clock Drawing Test (dCDT)	-	electronic pen	-
Garre-Olmo et al. [68]	2017	Digital Handwriting and Drawing Test	-	tablet and electronic pen	supervised
Ishikawa et al. [113]	2019	Digital Handwriting and Drawing Test:	-	tablet and electronic pen	-
Matusz et al. [114]	2022	Digital Clock Drawing Test (dCDT)	-	tablet and electronic pen	-
Müller, et al. [37]	2019	Digital Clock Drawing Test (dCDT)	-	tablet and electronic pen	-
Robens et al. [49]	2019	Digital Tree Drawing Test	-	tablet and electronic pen	-
Souillard-Mandar et al. [115]	2021	Digital Clock Drawing Test (dCDT)	-	web-based app on computer and electronic pen	supervised
Yu & Chang [48]	2019	Computerized Chinese Handwriting Task	-	tablet and electronic pen	supervised
Daily living task & Serious game					
Cabinio et al. [116]	2020	Smart Aging Serious Game (SASG)	4-20min	computer	-
Fukui et al. [50]	2015	Computerized Touch-Panel Games	< 5 min	computer	-
Gielis et al. [17,18]	2021a & b	Computer-based Klondike Solitaire	-	tablet	supervised
Harvey et al. [63]	2021	Computer-based functional skills assessment and training (CFSAT) and app version of Brief Assessment of Cognition (BACAPP)	-	computer with a touch screen or a mouse	supervised
Isernia et al. [19]	2021	Smart Aging Serious Game (SASG)	4-20 min	computer	-
Rapp et al. [51]	2018	SIMulation-Based Assessment of Cognition (SIMBAC)	-	tablet	supervised
Valladares-Rodriguez et al. [39]	2018	Episodix	-	computer	-
Vallejo et al. [117]	2017	Computer Simulation of Daily living Situations	-	touch-screen computer	supervised
