# Supplementary material for: Digital Cognitive Biomarker for Mild Cognitive Impairments and Dementia: A Systematic Review
Source: J Clin Med. 2022 Jul 19;11(14):4191. doi: 10.3390/jcm11144191 (PMC9320101; doi:10.3390/jcm11144191)
Supplement: Supplementary file 1 [file jcm-11-04191-s001.zip › Table S3_ MCI diagnosis.pdf]

Table S3. Diagnostic performance of digital cognitive biomarkers for mild cognitive impairments (MCI).

Author Names	Year	Digital biomarker	Sensitivity (%)	Specificity (%)	AUC	Comparison paper-and-pencil test	Sensitivity (%)	Specificity (%)	AUC
**Memory test**									
Alegret et al. [88]	2020	FACEmemory total score	73.40		0.77	-	-	-	-
Curiel et al. [64]	2016	MITSI-A2 + MITSI-B1	85.8	84.4	0.93	-	-	-	-
Junkkila et al. [52]	2012	CANTAB-PAL total errors adjusted			0.80	CERAD wordlist learning delayed recall	-	-	0.91
Liu et al. [89]	2021	MemTrax accuracy	72.00	84.00	0.84	MoCA-BJ	54.00	86.00	0.74
		MemTrax mean response time	42.00	66.00	0.53	-	-	-	-
		MemTrax composite score of accuracy and mean response time			0.71	-	-	-	-
Maki et al. [16]	2010	VSM test performance	70.00	76.00		Cube-copying test	25.00	-	-
Ramratan et al. [90]	2012	CRRST speed and accuracy	-	-	0.80				
Vacante et al. [53]	2013	total mean score on two computerized versions of TPT	70.00	76.20		paper-and-pencil version of TPT	30.00	95.50	-
van der Hoek et al. [92]	2019	MemTrax reaction speed	48.9	78.4	0.67	-	-	-	-
		MemTrax accuracy	43.2	93.3	0.66	-	-	-	-
**Test battery**									
Ahmed et al. [93]	2012	CANS-MCI composite z-score	89.0	73.0	0.87	MoCA	90.00	67.00	0.89
						ACE-R	90.00	67.00	0.82
Cho et al. [87]	2008	MCIS-J	95.8	100.0	-	MMSE	79.2	62.5	-
Darby et al. [96]	2002	Discriminant function using reaction time (RT) in SRT task and in matching RT task over trials, and the accuracy/speed ratio on trial 4 of 1-back task	80.00	95.00	-	-	-	-	-
Chin et al. [15]	2020	Inbrain CST total score	80.80	76.20	0.81	-	-	-	-
Dwoltzky et al. [55]	2003	NeuroTrax Mindstreams							
		memory all tests	-	-	0.65 - 0.84	WMS-III Logical Memory I & III; RAVLT Immediate Recall A & B & Total; RAVLT Delayed Recall; RAVLT Short Term Retention; RAVLT Recognition			0.632 - 0.885
		executive function all tests	-	-	0.70 - 0.81	CDT, TMT-A	-	-	0.660 - 0.680
		visual spatial all tests	-	-	0.77	WAIS-III Block Design	-	-	0.599

		verbal function all tests	-	-	0.72-0.82	Boston Naming Test, COWA FS, COWA A, COWA Animals, WAIS-III Similarities	-	-	0.553 - 0.768
		attention (Go-NoGo reaction time and SD of reaction time)	-	-	0.71-0.77	-	-	-	-
		information processing (high load and medium load in Staged Information Processing task)	-	-	0.69-0.78	-	-	-	-
Green et al. [99]	1994	ASC total score	83.00	96.00	-	-	-	-	-
Croppell et al. [83]	2019	BrainCheck overall performance	81.00	94.00	-	-	-	-	-
Gualtieri & Johnson [100]	2005	CNS Vital Signs				-	-	-	-
		composite score on memory domain	90.00	82.00	0.67	-	-	-	-
		composite score on psychomotor speed domain	90.00	77.00	0.68	-	-	-	-
		composite score on response time domain	90.00	64.00	0.66	-	-	-	-
		composite score on complex attention domain	90.00	65.00	0.74	-	-	-	-
		composite score on cognitive flexibility domain	90.00	76.00	0.69	-	-	-	-
Huang et al. [54]	2019	memory factor in Tablet-based cognitive assessments	-	-	0.74	MMSE	-	-	0.82
		reaction time factor Tablet-based cognitive assessments	-	-	0.72	-	-	-	-
Inoue et al. [77]	2005	Computerized Screening Test System total score	82	87	-	-	-	-	-
Maruff et al. [43]	2013	learning/working memory composite score	80.4	84.7	0.91	-	-	-	-
		attention/psychomotor composite	41.4	85.7	0.67	-	-	-	-
Memória et al. [71]	2014	z-score of the performance in Brazilian version of CANS-MCI	81.0	73.0	0.80	-	-	-	-
Possin et al. [65]	2018	performance in BHA	75.0	75.0	0.83	MoCA	56.00	75.00	0.74
		performance in BHA and Ecog12 survey	93.0	75.0	0.89	-	-	-	-
		performance in Complete BHA, including cognitive tests and BHS (Ecog-12 + additional 9 questions)	93.0	75.0	0.94	-	-	-	-
Rodríguez-Salgado [44]	2021	performance in BHA	92.0	83.0	0.94	MoCA	87.00	50.00	0.73
Ruano et al. [66]	2019	single performance on BoT tests at baseline	76.5	88.3	0.86	-	-	-	-
		performance on BoT in 12-month follow-up	100.0	73.0	0.94	-	-	-	-
Saxton et al. [35]	2009	performance in CAMCI	86.0	94.0	-	MMSE	45.00	80.00	-
Scharre et al. [72]	2017	eSAGE total score	63.0	81.0	0.78	-		-	-

Wouters et al. [78]	2009	CAMCOG-CAT performance	89	70	0.83	CAMCOG (full administration)	89	70	0.82
		CAMCOG-Plus-CAT performance	73	82	0.82	CAMCOG-Plus (full administration)	73	88	0.83
Ye et al. [104]	2022	BrainCheck overall score	86	83	0.84	-	-	-	-
Yu et al. [42]	2015	MoCA-CC total score	95.8	87.1	0.97	-	-	-	-
**Other single/multiple cognitive test**							-	-	
Cheah et al. [67]	2022	Rey-O copy performance	66.8	53.6	0.67	-	-	-	
		Rey-O immediate recall performance	73.6	88.5	0.87	-	-	-	
		Rey-O delay recall performance	84.7	90.5	0.91	-	-	-	
Garcia-Casal et al. [107]	2019	Emotion Recognition Task total correct answers	67.6	66.7	0.72	-	-	-	
		Emotion Recognition Task processing speed	56.3	87.7	0.75	-	-	-	
Wu et al. [45]	2017	e-CT age-corrected t-score of correct cancellations	71.2	76.9	0.81	K-T cancellation test	71.20	76.90	0.84
**Handwriting/drawing test**									
Binaco et al. [112]	2020	machine learning based on dCDT	Accuracy = 83.69%			-	-	-	-
Garre-Olmo et al. [68]	2017	Drawing tests (Discriminant function using kinematic and pressure features)							
		Cross pentagons	71.4	86.6	-	-	-	-	-
		Spiral	100.0	100.0	-	-	-	-	-
		3D house	84.0	93.6	-	-	-	-	-
		Clock Drawing Test	81.8	83.3	-	-	-	-	-
		Spontaneous	92.3	100.0	-	-	-	-	-
		Handwriting tests (Discriminant function using kinematic and pressure features)							
		Copied	91.6	94.1	-	-	-	-	-
		Dictated	100.0	100.0	-	-	-	-	-
Müller et al. [37]	2019	machine learning on dCDT features such as hybrid of time in air, time not printing, and dCDT score	85.4	77.5	0.89	-	-	-	-
Robens et al. [49]	2019	dTDT average velocity and pen-up stroke length	83.0	56.0	0.77	-	-	-	-
**Daily living task & Serious game**									
Cabinio et al. [116]	2020	SASG total score	84.4	75.5	0.88	MoCA	71.9	90.6	0.89
Fukui et al. [50]	2015	Flipping card game (completion time) (recent memory)	76.9	70.7	-	-	-	-	-

Gielis et al. [18]	2021b	machine learning using digital biomarkers of cognitive performance in Klondike Solitaire	77.8	88.9	0.90	-	-	-	-
Isernia et al. [19]	2021	SASG total score	77.0	77.3	0.82	MoCA	83.8	76.7	0.89
Rapp et al. [51]	2018	Total accuracy in SIMBAC	79.0	58.0	0.77	MMSE	Accuracy = 65%		
						composite score of RAVLT-Delayed recall, Boston Naming Test, Digit Span, Digit Symbol Coding, and TMT-B	Accuracy = 71%		

Abbreviations. ACE-R: Addenbrooke's Cognitive Examination-Revised; ASC: Assessment of Cognitive Skills; BHA: Brain Health Assessment; BoT: Brain on Track Self-applied Computerized Test; CAMCI: Computer Assessment of Mild Cognitive Impairment; CAMCOG: Cambridge Cognitive Examination; CAMCOG-CAT: Cambridge Cognitive Examination administered by Computerized Adaptive Testing; CANTAB: Cambridge Neuropsychological Test Automated Battery; CANS-MCI: Computer-Administered Neuropsychological Screen for Mild Cognitive Impairment; CERAD: The Consortium to Establish a Registry for Alzheimer's Disease; CNS Vital Signs: Computerized Neurocognitive Screening Battery; COWA: Controlled Oral Word Association Test; CRRST: Computerized Cued-Recall Retrieval Speed Test; dCDT: digital Clock Drawing Test; e-CT: electronic version of Cancellation Test; eSAGE: electronic version of Self-Administered Gerocognitive Examination; Inbrain CST: Inbrain Cognitive Screening Test; MCIS-J: Japanese version of Mild Cognitive Impairment Screen; MITSI-L: The Miami Test of Semantic Interference; MMSE: Mini-Mental State Examination; MoCA: The Montreal Cognitive Assessment; MoCA-BJ: Beijing version of The Montreal Cognitive Assessment; MoCA-CC: computerized tool for Computerized tool for Beijing version of The Montreal Cognitive Assessment (MoCA); PAL: Paired Associative Learning; RAVLT: Rey Auditory Verbal Learning Test; Rey-O: Rey–Osterrieth complex figure; SASG: Smart Aging Smart Game; SIMBAC: SIMulation-Based Assessment of Cognition; SRT: Simple Reaction Time; TMT-B: Trail-Making Test - Part B; TPT: The Placing Test; VSM: Visual Spatial Memory; WMS-III: Wechsler Memory Scale, 3rd edition.
