# Supplementary material for: Digital Cognitive Biomarker for Mild Cognitive Impairments and Dementia: A Systematic Review
Source: J Clin Med. 2022 Jul 19;11(14):4191. doi: 10.3390/jcm11144191 (PMC9320101; doi:10.3390/jcm11144191)
Supplement: Supplementary file 1 [file jcm-11-04191-s001.zip › Table S4_dementia diagnosis.pdf]

Table S4. Diagnostic performance of digital cognitive biomarkers for dementia.

Author Names	Year	Digital biomarker	Sensitivity (%)	Specificity (%)	AUC	Comparison paper-and-pencil test	Sensitivity (%)	Specificity (%)	AUC
**Memory test**									
Vacante et al. [53]	2013	total mean score on two computerized versions of TPT	88.9	92.9	-	Traditional paper-and-pencil version of TPT	88.9	95.5	-
**Test battery**									
Bissig et al. [76]	2020	SATURN total score	92.0	88.0	0.95	MoCA	-	-	-
Dougherty et al. [69]	2010	CST total score	99.0	95.0	0.99	MMSE	83.0	38.0	0.82
Memória et al. [71]	2014	z-score of the performance in Brazilian version of CANS-MCI	100.0	97.0	0.98	-	-	-	-
Fichman et al. [98]	2008	logistic regression using accuracy in Face Test, reaction time in Word Test, and reaction time in Forms with short-term memory test	91.8	93.6	-	-	-	-	-
Gualtieri & Johnson [100]	2005	CNS Vital Signs							
		composite score on memory domain	90.0	56.0	0.70	-	-	-	-
		composite score on psychomotor speed domain	90.0	-	0.57	-	-	-	-
		composite score on response time domain	90.0	94.0	0.64	-	-	-	-
		composite score on complex attention domain	90.0	-	0.54	-	-	-	-
		composite score on cognitive flexibility domain	90.0	-	0.61	-	-	-	-
Inoue et al. [73]	2009	Delay recall	69.0	95.0	0.84	-	-	-	-
		Temporal orientation	69.0	87.0	0.80	-	-	-	-
		Computerized Screening Test Battery total score	97.0	85.0	0.93	-	-	-	-
Maruff et al. [43]	2013	Learning/working memory composite score	100.0	84.7	0.99	-	-	-	-
		Attention/psychomotor composite	52.9	85.7	0.73	-	-	-	-
Possin et al. [65]	2018	BHA performance	100.0	75.0	0.95	MoCA	98.0	75.0	0.92
		performance in BHA and Ecog12 survey	100.0	75.0	> 0.99	-	-	-	-

		performance in Complete BHA, including cognitive tests and BHS (Ecog-12 + additional 9 questions)	100.0	75.0	> 0.99	-	-	-	-
Rodríguez-Salgado [44]	2021	BHA performance	96.0	98.0	0.98	MoCA	82.0	96.0	0.97
Scanlon et al. [70]	2016	CCS total score	94.0	60.0	0.94	MoCA	95.0	100.0	0.99
Scharre et al. [72]	2017	eSAGE total score	95.0	100.0	0.99	-	-	-	-
Ye et al. [104]	2022	BrainCheck overall score	88.0	94.0	0.95	-		-	-
**Other single/multiple cognitive tests**									
Angelillo et al. [106]	2019	machine learning model on the handwriting movements in three matrices of the attentional matrices test	86.1	82.8	0.87	-	-	-	-
Cheah et al. [67]	2022	Rey-O copy performance	62.7	90.0	0.76	-	-	-	-
		Rey-O immediate recall performance	82.7	94.7	0.95	-	-	-	-
		Rey-O delay recall performance	82.0	95.3	0.94	-	-	-	-
Garcia-Casal et al. [107]	2019	total correct answers in emotion recognition score task	76.1	75.0	0.79	-	-	-	-
		processing speed scores	77.5	78.6	0.83	-	-	-	-
Wu et al. [45]	2017	age-corrected t-score of correct cancellations on e-CT	86.1	91.7	0.92	K-T cancellation test	86.1	94.4	0.86
**Handwriting/drawing test**									
Binaco et al. [112]	2020	machine learning on dCDT	Accuracy = 91.4%			-	-	-	-
Davoudi et al. [57]	2020	machine learning on dCDT copy & command	97.7	71.4	0.92	-	-	-	-
Müller et al. [37]	2019	dCDT parameters (hybrid of time in air and dCDT score)	91.5	94.2	0.97	CERAD neuropsychological battery total score	88.7	94.9	0.98
Robens et al. [49]	2019	dTDT (no. of colors used, No. of line widths used, and average velocity)	82.0	86.0	0.90	-	-	-	-
**Daily living task & Serious game**									
Rapp et al. [51]	2018	SIMBAC total accuracy	86.0	75.0	0.97	MMSE	Accuracy = 83%		

		Composite score of RAVLT-Delayed recall, Boston Naming Test, Digit Span, Digit Symbol Coding, and TMT-B	Accuracy = 91%

Abbreviations. BHA: Brain Health Assessment; BHS: Brain Health Survey; CANS-MCI: Computer-Administered Neuropsychological Screen for Mild Cognitive Impairment; CCS: Computerized Cognitive Screening; CERAD: The Consortium to Establish a Registry for Alzheimer's Disease CNS Vital Signs: Computerized Neurocognitive Screening Battery; CST: Computer Self-Test dCDT: digital Clock Drawing Test; e-CT: electronic version of Cancellation Test; eSAGE: electronic version of Self-Administered Gerocognitive Examination; MMSE: Mini-Mental State Examination; MoCA: The Montreal Cognitive Assessment; RAVLT: Rey Auditory Verbal Learning Test; Rey-O: Rey–Osterrieth complex figure; SATURN: Self-Administered Tasks Uncovering Risk of Neurodegeneration; SIMBAC: SIMulation-Based Assessment of Cognition; TMT-B: Trail-Making Test - Part B; TPT: The Placing Test.
