# Supplementary material for: Digital Cognitive Biomarker for Mild Cognitive Impairments and Dementia: A Systematic Review
Source: J Clin Med. 2022 Jul 19;11(14):4191. doi: 10.3390/jcm11144191 (PMC9320101; doi:10.3390/jcm11144191)
Supplement: Supplementary file 1 [file jcm-11-04191-s001.zip › Table S5_CI diagnosis.pdf]

Table S5. Diagnostic performance of digital cognitive biomarkers for cognitive impairments (CI).

Author Names	Year	Digital biomarker	Sensitivity (%)	Specificity (%)	AUC	Comparison paper-and-pencil test	Sensitivity (%)	Specificity (%)	AUC
**Memory test**									
Rafii et al. [74]	2011	MCIS performance	91.8	72.0	0.89	-	-	-	-
**Test battery**									
Chan et al. [95]	2020	regression model (score of the 5-word delayed recognition test, the clock-setting test, and the story test, and completion time of the 5-word delayed recognition test and clock-setting test)	81.0	80.0	0.87	-	-	-	-
Chin et al. [15]	2020	Inbrain CST total score	82.4	82.8	0.93	-	-	-	-
Cho et al. [59]	2002	Memory impairment screen subset	70.7	89.3	-	-	-	-	-
		CDST all subsets	75.6	94.2	-	-	-	-	-
Rodríguez-Salgado [44]	2021	BHA performance	91.0	85.0	0.95	MoCA	85	63	0.83
Scharre et al. [72]	2017	eSAGE total score	71.0	90.0	0.88	Paper-and-pencil version of SAGE	69	88	0.83
Takahashi et al. [61]	2021	z-score of CompBased-CAT	81.0	77.0	0.85	-	-	-	-
Wong et al. [60]	2017	CoCoSC Total score	78.0	69.0	0.78	-	-	-	-
**Other single/multiple cognitive tests**									
Kokubo et al. [58]	2018	TMT total score	82.0	91.0	0.93	MMSE	97	83	0.90
		TMT total score and age	97.0	92.0	0.97	-			
Wu et al. [45]	2017	age-corrected t-score of correct cancellations on e-CT	77.0	80.6	0.77	K-T cancellation test	76.5	80.6	0.87
**Handwriting/drawing test**									
Amini et al. [111]	2021	machine learning on dCDT command & copy, education level, and age	86.9	84.5	0.92	-	-	-	-
Garre-Olmo et al. [68]	2017	Drawing tests (Discriminant function using kinematic and pressure features)							
		Cross pentagons	89.70	100.00	-	-	-	-	-
		Spiral	85.00	91.60	-	-	-	-	-
		3D house	81.40	100.00	-	-	-	-	-
		Clock Drawing Test	79.50	100.00	-	-	-	-	-

		Handwriting (Discriminant function using kinematic and pressure features)							
		Spontaneous	84.2	78.5	-	-	-	-	-
		Copied	81.5	76.9	-	-	-	-	-
		Dictated	76.1	70.0	-	-	-	-	-
Robens et al. [49]	2019	regression model using gender, age, education, strokes per minute, volatile motion, line width changes, and entropy in dTDT	77.0	78.0	0.84	-	-	-	-
Souillard-Mandar et al. [115]	2021	machine learning based on dCDT	74.0	90.0	0.89	MMSE	67	90	-
**Daily living task**									
Rapp et al. [51]	2018	SIMBAC total accuracy	70.0	82.0	0.84	-	-	-	-

Abbreviations. BHA: Brain Health Assessment; CoCoSc: Computerized Cognitive Screen; CompBased-CAT: Computer-Based Cognitive Assessment Tool; dCDT: digital Clock Drawing Test; e-CT: electronic version of Cancellation Test; eSAGE: electronic version of Self-Administered Gerocognitive Examination; Inbrain CST: Inbrain Cognitive Screening Test; MCIS: Mild Cognitive Impairment Screen; MMSE: Mini-Mental State Examination; MoCA: The Montreal Cognitive Assessment; SAGE: Self-Administered Gerocognitive Examination; SIMBAC: SIMulation-Based Assessment of Cognition; TMT: Trail-Making Test.
