# Supplementary material for: Digital Cognitive Biomarker for Mild Cognitive Impairments and Dementia: A Systematic Review
Source: J Clin Med. 2022 Jul 19;11(14):4191. doi: 10.3390/jcm11144191 (PMC9320101; doi:10.3390/jcm11144191)
Supplement: Supplementary file 1 [file jcm-11-04191-s001.zip › Table S6_risk assessment.pdf]

Table S6. Quality assessment based on Newcastle–Ottawa Quality Assessment Scale Criteria.

		Selection				Comparability		Exposure			
**Author**	**Year**	**Is the case definition adequate**	**Representativeness of the cases**	**Selection of Controls**	**Definition of Controls**	**Age**	**Education**	**Ascertainment of exposure**	**Same method of ascertainment for cases and controls**	**Non-Response rate**	**Total score**
**Memory test**											
Alegret et al. [88]	2020	1	1	1	1	1	1	1	1	1	9
Curiel et al. [64]	2016	1	1	1	1	1	1	1	1	1	9
Junkkila et al. [52]	2012	1	1	1	1	1	1	1	0	1	8
Liu et al. [89]	2021	1	1	1	1	1	1	1	1	1	9
Maki et al. [16]	2010	1	1	1	1	1	1	0	0	1	7
Rafii et al. [74]	2011	1	1	1	1	1	1	1	1	0	8
Ramratan et al. [90]	2012	1	1	1	1	1	1	1	1	0	8
Troyer et al. [91]	2016	1	1	1	1	1	1	1	1	1	9
Vacante et al. [53]	2013	1	1	1	1	1	1	1	1	1	9
van der Hoek et al. [92]	2019	1	1	1	1	1	1	1	1	1	9
**Test battery**											
Ahmed et al. [93]	2012	1	1	1	1	1	1	1	1	0	8
Bissig et al. [76]	2020	1	1	1	1	0	0	1	0	1	6
Cerino et al. [94]	2021	1	1	1	1	1	1	1	1	1	9
Chan et al. [95]	2020	1	1	1	1	1	1	1	0	0	7
Chin et al. [15]	2020	1	1	1	1	1	1	1	0	1	8
Cho et al. [59]	2002	1	1	1	1	1	1	0	0	1	7
Cho et al. [87]	2008	1	1	1	1	1	1	1	1	1	9
Darby et al. [96]	2002	1	1	1	1	1	1	1	1	1	9
Dorociak et al. [97]	2021	1	1	1	1	1	1	1	1	1	9
Dougherty et al. [69]	2010	1	1	1	1	1	1	1	1	1	9
Dwolatzky et al. [55]	2003	1	1	1	1	1	0	1	1	0	7
Égerházi et al. [41]	2007	1	1	1	1	1	1	1	0	1	8
Fichman et al. [98]	2008	1	1	1	1	1	1	1	0	1	8

Green et al. [99]	1994	1	1	1	1	1	1	1	0	1	8
Groppell et al. [83]	2019	1	1	1	1	0	0	1	1	1	7
Gualtieri & Johnson [100]	2005	1	1	1	1	1	1	1	0	1	8
Huang et al. [54]	2019	1	1	1	1	1	1	1	1	0	8
Inoue et al. [77]	2005	1	1	1	1	0	0	0	0	1	5
Inoue et al. [73]	2009	1	1	1	1	1	0	0	0	1	6
Maruff et al. [43]	2013	1	1	1	1	1	0	1	1	1	8
Memória et al. [71]	2014	1	1	1	1	1	1	1	1	1	9
Mundt et al. [101]	2007	1	1	1	1	1	1	1	1	1	9
Possin et al. [65]	2018	1	1	1	1	1	1	1	1	1	9
Rodríguez-Salgado [44]	2021	1	1	1	1	1	1	1	1	1	9
Ruano et al. [66]	2019	1	1	1	1	1	1	0	1	1	8
Ruano et al. [102]	2016	1	1	1	1	1	1	0	0	1	7
Saxton et al. [35]	2009	1	1	1	1	1	1	1	1	1	9
Scanlon et al. [70]	2016	1	1	1	1	1	1	1	0	0	7
Scharre et al. [72]	2017	1	1	1	1	1	1	1	1	1	9
Takahashi et al. [61]	2021	1	1	1	1	0	0	1	1	1	7
Veroff et al. [103]	1991	1	1	1	1	1	1	1	1	1	9
Vyshedskiy et al. [62]	2022	1	1	1	1	1	1	1	1	1	9
Wong et al. [60]	2017	1	1	1	1	0	1	1	1	1	8
Wouters et al. [78]	2009	1	1	1	1	0	0	0	0	1	5
Ye et al. [104]	2022	1	1	1	1	1	1	1	1	0	8
Yu et al. [42]	2015	1	1	1	1	1	1	1	1	1	9
Zhang et al. [105]	2017	1	1	1	1	1	1	1	1	1	9
**Other single/multiple cognitive tests**											
Angelillo et al. [106]	2019	1	1	1	1	1	1	1	1	1	9
Bonney et al. [46]	2006	1	1	1	1	1	1	0	1	0	7
Cheah et al. [67]	2022	1	1	0	1	1	1	1	1	1	8
Chen et al. [56]	2017	1	1	1	1	1	1	1	1	1	9

García-Casal et al. [107]	2019	1	1	1	1	1	1	1	1	1	9
Kalová et al. [108]	2005	1	1	0	1	1	1	1	1	1	8
Kokubo et al. [58]	2018	1	1	1	1	1	1	0	1	1	8
Lunardini et al. [109]	2020	1	1	1	1	1	1	1	1	1	9
Mollica et al. [110]	2017	1	1	1	1	1	1	1	0	1	8
Simfukwe et al. [47]	2021	1	1	1	1	0	0	1	1	0	6
Wu et al. [45]	2017	1	1	1	1	1	1	1	1	1	9
Zhou et al. [86]	2017	1	1	1	1	1	1	1	1	1	9
**Handwriting/drawing test**											
Amini et al. [111]	2021	1	1	1	1	1	1	1	1	1	9
Binaco et al. [112]	2020	1	1	1	1	1	1	1	1	1	9
Davoudi et al. [57]	2020	1	1	1	1	0	0	1	0	1	6
Garre-Olmo et al. [68]	2017	1	1	1	1	1	0	1	1	1	8
Ishikawa et al. [113]	2019	1	1	1	1	1	0	1	0	1	7
Matusz et al. [114]	2022	1	1	1	1	1	1	1	1	1	9
Müller, et al. [37]	2019	1	1	1	1	1	1	0	0	1	7
Robens et al. [49]	2019	1	1	1	1	1	1	1	0	1	8
Souillard-Mandar et al. [115]	2021	1	1	1	1	1	1	1	1	1	9
Yu & Chang [48]	2019	1	1	1	1	1	1	1	1	1	9
**Daily living task & Serious game**											
Cabinio et al. [116]	2020	1	1	1	1	1	1	1	1	1	9
Fukui et al. [50]	2015	1	1	0	1	1	1	1	0	1	7
Gielis et al. [17,18]	2021a & b	1	1	1	1	1	0	1	0	1	7
Harvey et al. [63]	2021	1	1	1	1	1	0	1	1	1	8
Isernia et al. [19]	2021	1	1	1	1	1	1	1	1	0	8
Rapp et al. [51]	2018	1	1	1	1	1	1	1	1	1	9
Valladares-Rodriguez et al. [39]	2018b	1	1	1	1	0	0	1	0	1	6

Vallejo et al. [117]	2017	1	1	1	1	0	1	1	0	1	7
