# Supplementary figures and images for: Digital Cognitive Biomarker for Mild Cognitive Impairments and Dementia: A Systematic Review
Source: J Clin Med. 2022 Jul 19;11(14):4191. doi: 10.3390/jcm11144191 (PMC9320101; doi:10.3390/jcm11144191)

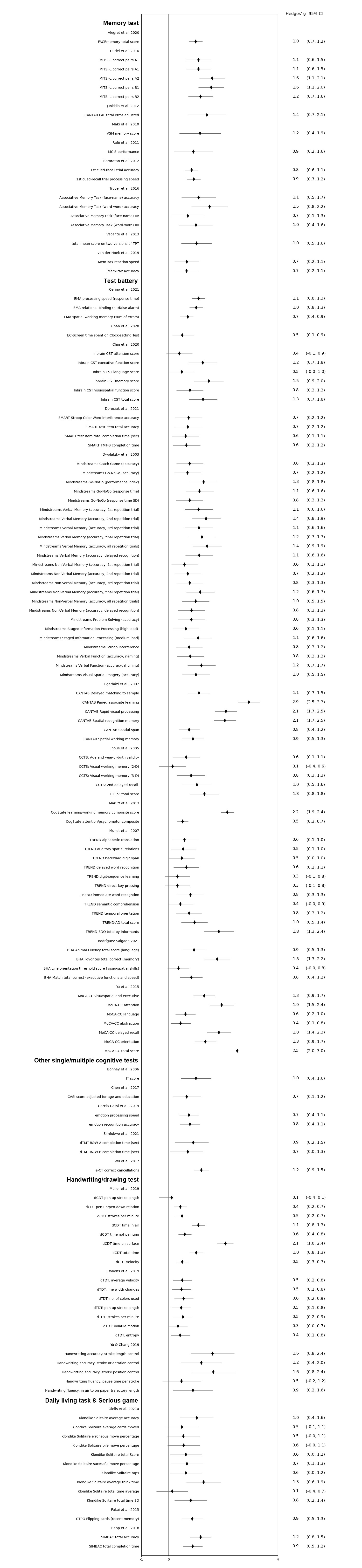

Supplement: Supplementary file 1 [file jcm-11-04191-s001.zip › Figure S1.jpg]

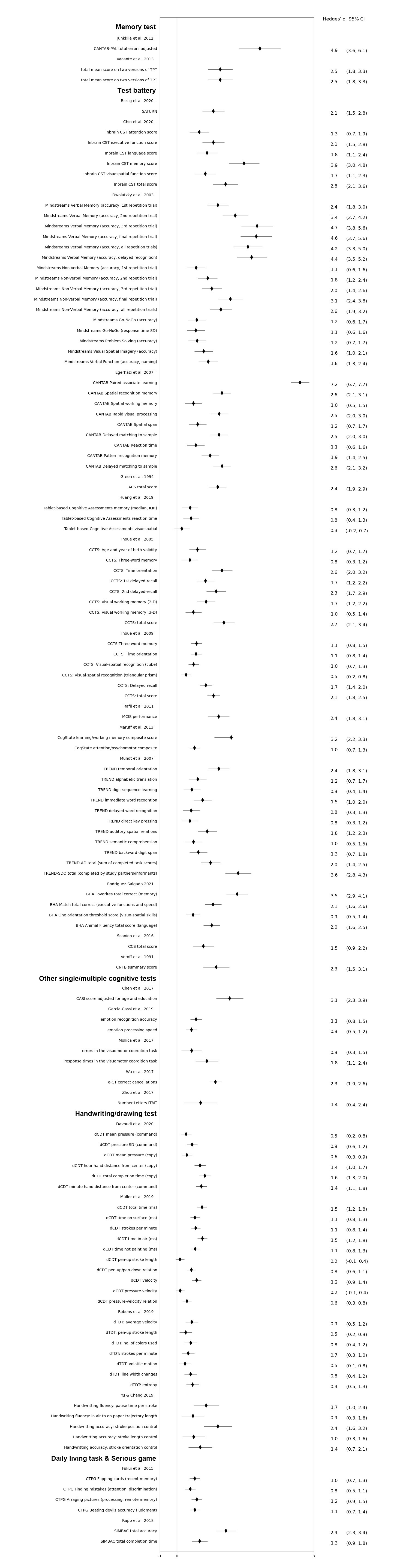

Supplement: Supplementary file 1 [file jcm-11-04191-s001.zip › Figure S2.jpg]

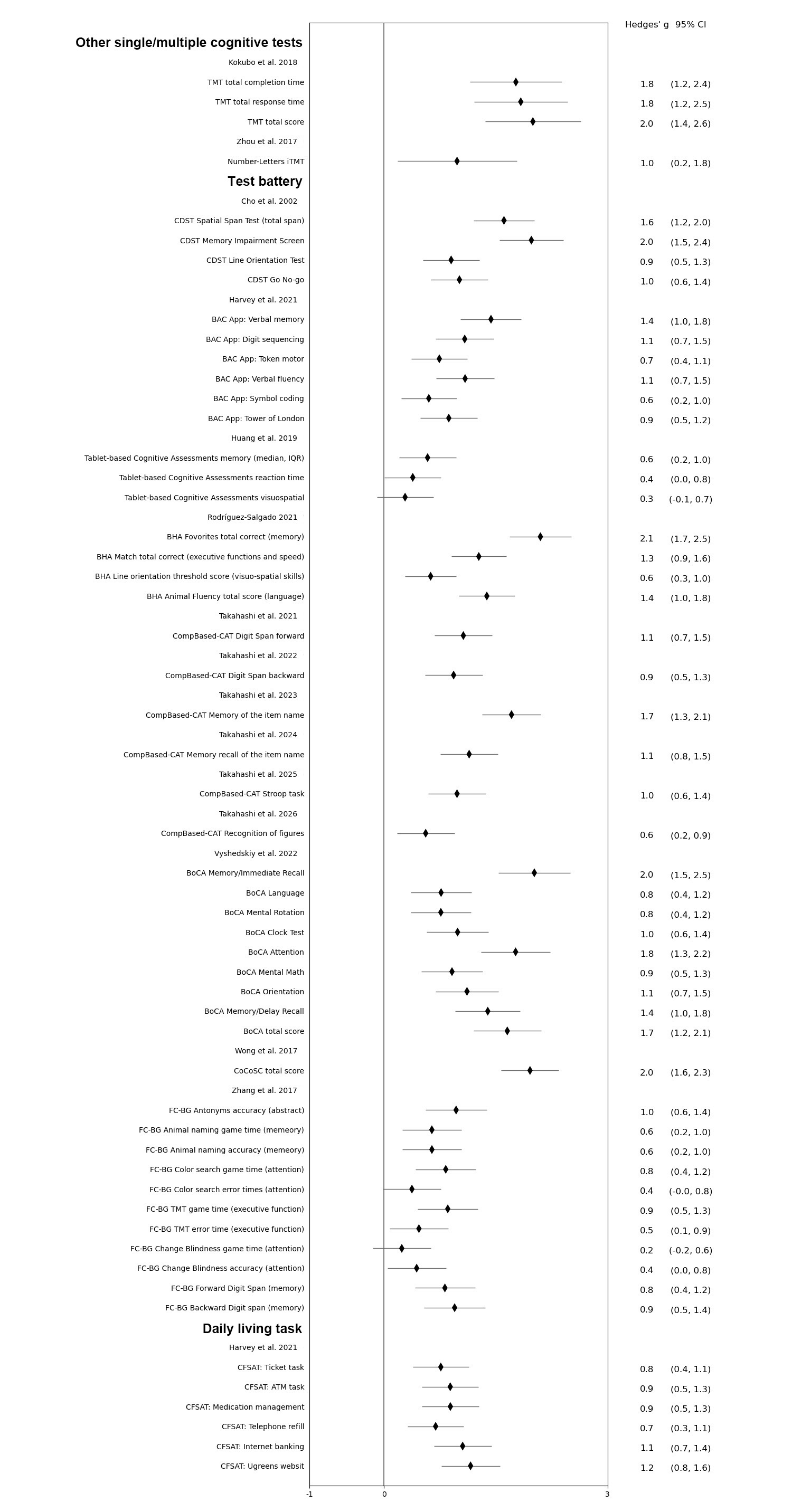

Supplement: Supplementary file 1 [file jcm-11-04191-s001.zip › Figure S3.jpg]
